# Nanoparticle‐Modified Nanofibers Induce Ferroptosis and Stimulate Antitumor Immunity for Melanoma Therapy

**DOI:** 10.1002/advs.202508753

**Published:** 2025-10-20

**Authors:** Mingyang Li, Rongrong Li, Baotong Xu, Yuhan Lian, Chenyu Yang, Jingwen Li, Junhao Liang, Linhan Ding, Dongsheng Zhang, Jing Guo, Xiao Fu

**Affiliations:** ^1^ Department of Stomatology Shandong Provincial Hospital Affiliated to Shandong First Medical University No.324 Jingwuweiqi Road, Huaiyin District Jinan Shandong Province 250021 P. R. China; ^2^ Department of Dermatology Shandong Provincial Hospital Affiliated to Shandong First Medical University No.324 Jingwuweiqi Road, Huaiyin District Jinan Shandong Province 250021 P. R. China; ^3^ School of Stomatology Shandong First Medical University and Shandong Academy of Medical Sciences Jinan Shandong 250021 P. R. China

**Keywords:** ferroptosis, immunotherapy, melanoma, metal‐polyphenol network, nanofiber

## Abstract

Melanoma, a highly aggressive and therapy‐resistant cutaneous malignancy, presents formidable clinical challenges owing to its propensity for early metastasis and frequent postoperative recurrence. Here, a nanofiber‐based drug delivery system (5F/STFM/R‐ES) is developed that synergistically induces ferroptosis and potentiates immunotherapy to inhibit melanoma growth and reduce the risk of metastasis and recurrence. To this end, mesoporous silica nanoparticles encapsulating resiquimod (R848) are surface‐coated with Fe/Mn‐based metal‐polyphenol networks (TFM) to form STFM/R. Subsequently, STFM/R is electrospun into 5‐fluorouracil‐loaded nanofibers. When applied to the tumor site, 5‐fluorouracil and STFM/R are released. Following the degradation of STFM/R, the release of Fe^2^⁺ triggered ferroptosis through iron overload, while the simultaneous liberation of Mn^2^⁺ synergizes with R848 to activate the cGAS‐STING signaling pathway. This multifunctional strategy promotes the maturation of dendritic cells and repolarizes tumor‐associated macrophages from M2 to M1. The in vivo and in vitro evaluations reveal that 5F/STFM/R‐ES significantly improves delivery efficacy, remodels the immunosuppressive tumor microenvironment, and enhances the therapeutic efficacy of chemotherapeutics. The localized delivery strategy of the platform minimizes systemic toxicity while achieving combinatorial therapeutic effects, highlighting its potential for personalized melanoma therapy.

## Graphical abstract

This study presents a nanoparticle‐embedded nanofiber (5F/STFM/R‐ES) to inhibit melanoma growth and reduce the risk of metastasis and recurrence. The drug delivery system induces ferroptosis and potentiates immunotherapy through resiquimod (R848) and Fe/Mn‐based metal‐polyphenol networks (TFM). The localized delivery strategy of the platform minimizes systemic toxicity while achieving combinatorial therapeutic effects, highlighting its potential for personalized melanoma therapy.

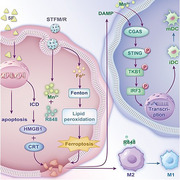

## 1 Introduction

Melanoma is a highly aggressive cancer with increasing incidence.^[^ ^1^ ^]^ It comprises only 1% of skin cancer cases but contributes to over 80% of fatalities.^[^ ^2^, ^3^ ^]^ Although the clinical translation of molecularly targeted agents and immunotherapeutic regimens has led to substantial advances in melanoma therapy, a number of patients still fail to attain optimal therapeutic outcomes, owing to issues such as treatment resistance and recurrence.^[^ ^4^, ^5^ ^]^

The development of immunotherapy has opened new avenues for cancer treatment, and the field of cancer immunotherapy is experiencing continuous rejuvenation.^[^ ^6^ ^]^ The purpose of immunotherapy is to activate the host immune system to recognize and kill the malignant tumors.^[^ ^7^ ^]^ Several types of cancers, especially melanoma, have demonstrated a prolonged clinical response to immunotherapy.^[^ ^8^ ^]^ Various immunotherapies strategies such as cancer vaccines, adoptive cell transfer (ACTs), immune checkpoint inhibitors (ICIs), and oncolytic virus therapy (OVTs) have shown promising clinical outcomes.^[^ ^9^, ^10^ ^]^ As a novel and critical innate immune signaling pathway, the cGAS‐STING pathway is triggered by nucleic acids and interacts with other immune responses, thereby playing an essential role in cancer immunoregulation.^[^ ^11^ ^]^ cGAS efficiently identifies all free cytoplasmic DNA originating from both self‐and foreign sources such as viruses, dead cells, tumor cells, and microorganisms.^[^ ^12^ ^]^ The cGAS synthesizes cyclic guanosine monophosphate‐adenosine monophosphate (cGAMP) using ATP and GTP within the cytoplasm as raw materials.^[^ ^13^ ^]^ Afterward, STING bound to cGAMP moves from the ER to the Golgi apparatus. During migration, STNG could recruit and activate TBK1 and IKK kinases, which in turn activate downstream IRF3 and NF‐κB, thereby inducing increasing expression of type I interferon (type I IFN) and a range of inflammatory factors that initiate the innate immune response.^[^ ^14^ ^]^ Metal cations, such as manganese (Mn^2+^) and zinc (Zn^2+^), can activate the cGAS‐STING signaling pathway, demonstrating their potential as agents for immune activation.^[^ ^15^ ^]^ Moreover, immune activation is regulated by Toll‐like receptors (TLRs), particularly TLR7/8.^[^ ^16^ ^]^ Activation of TLR7/8 can lead to the upregulation of co‐stimulatory factor expression in dendritic cells (DCs) and induce a shift in macrophage polarization from the M2 to the M1 phenotype. This process can work synergistically with cGAS‐STING agonists to enhance antitumor immunity. Among TLR agonists, the TLR7/8 agonist resiquimod (R848) has been employed as an immunoadjuvant for the maturation of DCs and the subsequent expansion of cytotoxic T lymphocytes with the secretion of multiple cytokines. In the present study, we used Mn^2+^ and R848 to enhance the efficacy of immunotherapy for melanoma treatment.

Ferroptosis is an iron‐dependent form of cell death driven by lipid peroxidation in cellular membranes and is considered a potential therapeutic strategy against cancers, including melanoma.^[^ ^17^, ^18^, ^19^ ^]^ Excessive iron ions in cancer cells lead to a violent Fenton reaction and subsequently catalyze the lipid peroxidation of polyunsaturated fatty acids, which finally induces cell death.^[^ ^20^ ^]^ A great number of investigations have demonstrated the critical role of ferroptosis in melanoma treatment.^[^ ^21^ ^]^ Lipid metabolism reprogramming in melanoma cells induces the accumulation of polyunsaturated fatty acids, which makes melanoma sensitive to ferroptosis; higher ferroptosis levels in melanoma not only inhibit cancer progression, but also prevent the metastasis of cancer cells.^[^ ^22^ ^]^ Ferroptosis is also known as immunogenic cell death (ICD).^[^ ^23^ ^]^ During ferroptosis, the release of immunostimulatory signals, notably damage‐associated molecular pattern signals such as High mobility group box‐1 protein (HMGB1), calreticulin, DNA, and ATP, can effectively trigger an immune response.^[^ ^24^ ^]^ In contrast, the release of IFN‐γ from CD8^+^T cells during immunotherapy could inhibit the function of System Xc‐, which could further enhance the ferroptosis level in cancer cells.^[^ ^25^ ^]^

To stimulate ferroptosis and the innate immune response in melanoma tissues, we used Mn^2+^, Fe^2+^ and tannic acid to synthesize metal‐polyphenol networks (TFM). Phenolic drugs serve as cytotoxic anticancer agents, showing great potential for inducing apoptosis, decreasing cell proliferation, and addressing multiple aspects of cancer progression, such as growth, angiogenesis, and metastasis.^[^ ^25^, ^26^ ^]^ Although stability and targeted delivery are critical for the biological activity of polyphenols, their bioavailability is a major issue in their use as cancer treatment agents; a promising way to solve this problem is through nano‐formulated polyphenols.^[^ ^27^ ^]^ The polyphenols interact with metal ions through metal chelation.^[^ ^28^ ^]^ Thus, the interaction between polyphenolic compounds and metal ions can lead to the formation of self‐assembled metal phenolic networks, which are a type of supramolecular amorphous structure.^[^ ^29^ ^]^ Depending on the acidic environment at the tumor site, TFM can simultaneously release Mn^2+^ and Fe^2+^, which are responsible for the activation of the immune response and ferroptosis, respectively. Because of their expansive surface area and robust loading capability, silica nanoparticles (MSN) are ideal carriers for drug delivery. In this study, we coated TFM onto the surface of drug‐encapsulated MSN to realize the delivery of TFM and R848.

The process of drug delivery is obstructed by various complex physiological barriers in vivo.^[^ ^30^ ^]^ To enhance the efficacy of drug delivery to melanoma, a variety of carriers—including liposomes, polymer nanoparticles, inorganic nanoparticles, and other systems—have been developed and are extensively utilized in melanoma treatment.^[^ ^31^ ^]^ These carriers have been shown to improve therapeutic outcomes.^[^ ^32^ ^]^ Nonetheless, significant challenges persist in the clinical application of nanosystems, primarily due to complex physiological and physical barriers, as well as concerns regarding patient adverse reactions, safety, and efficacy.^[^ ^33^ ^]^ It is challenging to resolve the low bioavailability, unstable targeting effects, and significant side effects of intravenous administration.^[^ ^34^ ^]^ Direct delivery of drugs to the tumor site could enhance local drug concentration while minimizing systemic exposure and related side effects. Compared with traditional nanocarriers, nanofiber nanocarriers, especially electrospinning, show great advantages such as high surface area, excellent permeability, easy incorporation of drugs, flexible material selection, and low fabrication costs, which make it easy to adjust structural design parameters such as morphology and surface area by adjusting the processing conditions to achieve the best drug loading effect.^[^ ^35^, ^36^ ^]^ In this study, we applied polylactic‐co‐glycolic acid (PLGA) electrospinning as a carrier for MSN to achieve local administration in melanoma treatment.

**Scheme 1:**
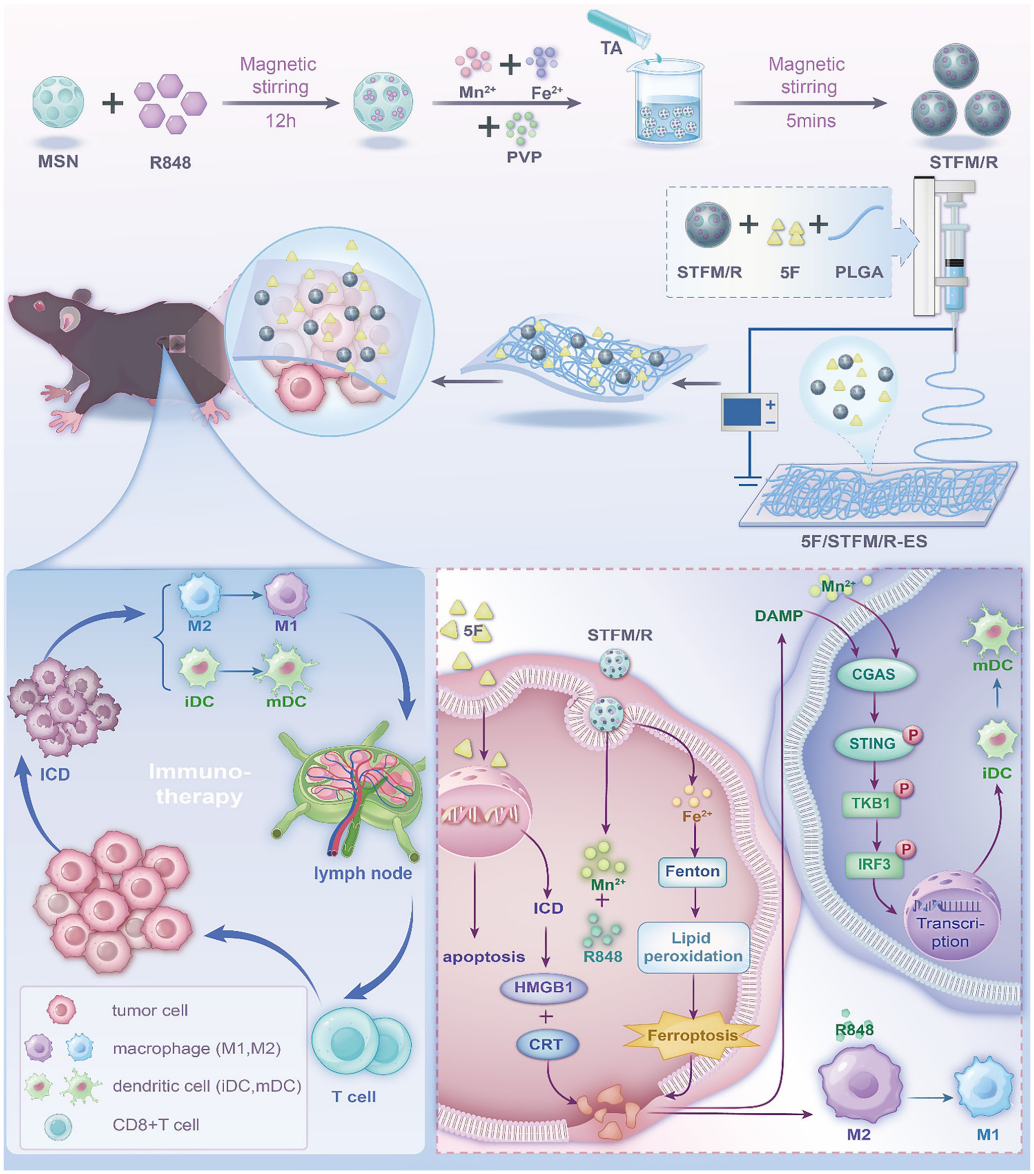
Schematic of the mechanism of 5F/STFM/R‐ES in treating melanoma.

Here, we developed a tumor‐specific drug delivery system using electrospinning technology for local chemotherapy, immunotherapy, and ferroptosis therapy. Mn^2+^, Fe^2+^ and tannic acid were applied to self‐assemble metal phenolic networks (TFM) and further coated on the surface of R848 loaded MSN.^[^ ^37^, ^38^ ^]^ Finally, STFM/R and chemotherapeutic 5‐fluorouracil (5‐FU) were embedded into nanofibers via electrospinning (5F/STFM/R‐ES) with poly (lactic‐co‐glycolic acid) (PLGA). This design allows controlled drug release within tumor tissues, maintains local drug concentration, and reduces systemic side effects. The 5‐FU released by electrospinning can directly kill cancer cells and induce ICD. Then, Fe^2+^ released from STFM/R enhances ferroptosis in melanoma cells. Mn^2+^ and R848 in STFM/R can activate the antitumor immune response and remodel the microenvironment. In this way, we simultaneously boosted the effect of ferroptosis therapy and immunotherapy for melanoma treatment, enhanced the cytotoxicity of ICD‐inducing drugs (5‐FU), and demonstrated potent tumor growth inhibition, providing new hope for cancer management (**Scheme** **1**).

## 2 Results

### 2.1 Preparation and Characterization of 5F/STFM/R‐ES

**Figure 1:**
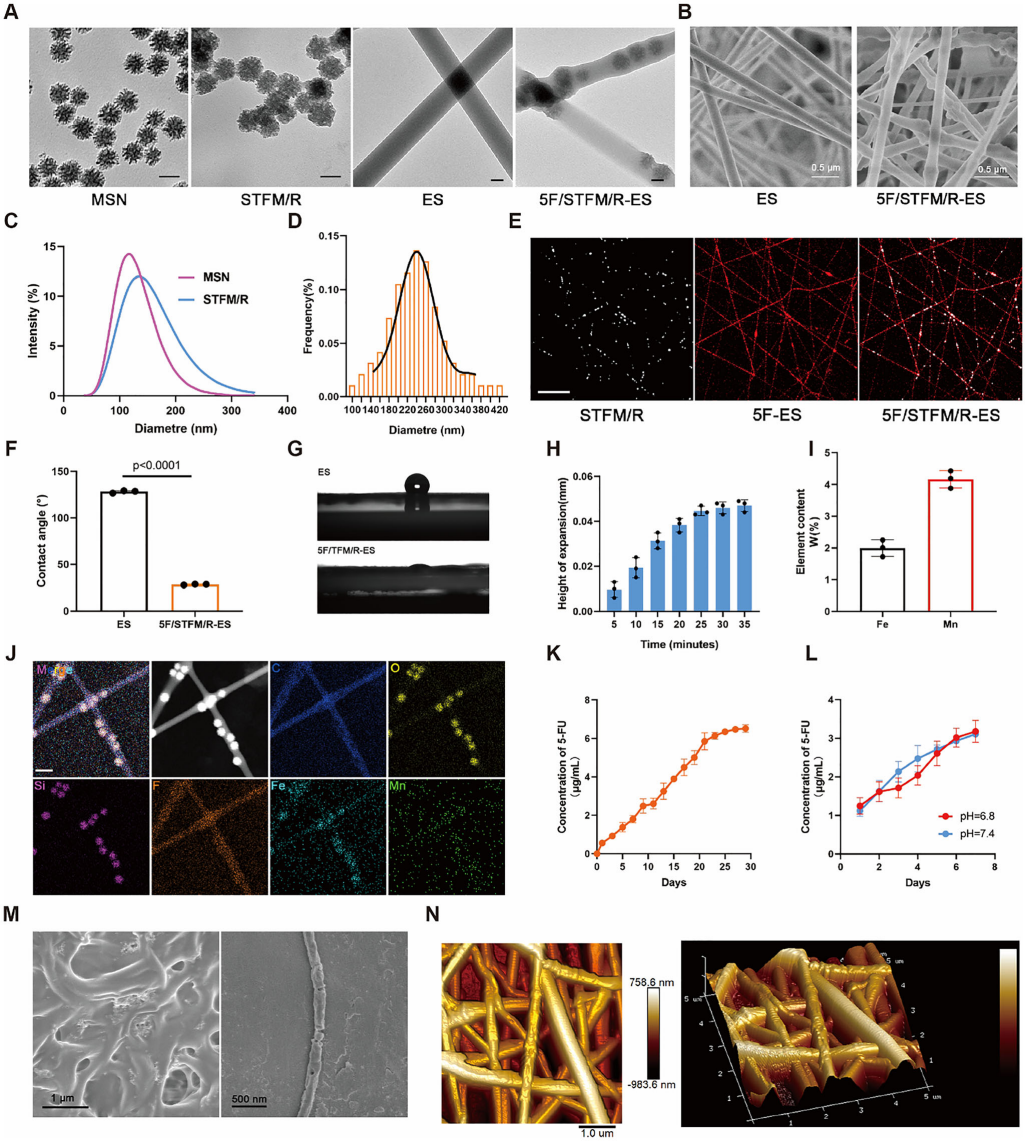
Characterization of 5F/STFM/R‐ES. A) Representative TEM images of MSN, STFM, ES (unloaded electrospinning nanofibers) and 5F/STFM/R‐ES (scale bar equals 100 nm). B) SEM images of ES and 5F/STFM/R‐ES. C) Distribution of diameter of MSN and STFM detected by DLS. D) The diameter distribution of 5F/STFM/R‐ES analyzed from TEM images of 5F/STFM/R‐ES. E) Representative images of 5F/STFM/R‐ES taken by LSCM (white for STFM/R and red for 5F‐ES) (scale bar equals 20 µm). F,G) Contact angle of 5F/STFM/R‐ES. H) Height of expansion analysis of 5F/STFM/R‐ES (n=3 experiments). I) Element content in 5F/STFM/R‐ES measured by ICP (n=3 experiments). J) High‐angle annular dark‐field scanning TEM image of 5F/STFM/R‐ES and the corresponding element mapping images (scale bar equals 500 nm). K,L) In vitro drug release analysis of 5F/STFM/R‐ES (n=3 experiments). M) Representative SEM images showing the morphology of 5F/STFM/R‐ES after drug release. N) Representative AFM images of 5F/STFM/R‐ES. Data are presented as mean ± SEM. *p* values were determined by 2‐tailed Student's *t*‐test.

R848 was encapsulated in MSN to form S/R (MSN loaded with R848), and STFM/R (S/R coated with TFM) was synthesized simultaneously, following the methods described in our previous study.^[^ ^39^ ^]^ PLGA is widely recognized for its biodegradability, low immunogenicity, biocompatibility, and mechanical strength. By manipulating the spinning material ratio and structural properties, PLGA nanofibers could reduce the pre‐implantation burst release, which was approved by the FDA as a carrier for drug release. These properties of PLGA enabled prolonged and delayed drug release and facilitated long‐term drug delivery. Therefore, STFM/R and 5‐FU were encapsulated in PLGA‐based nanofibers (5F/STFM/R‐ES) subsequently. Following synthesis, 5F/STFM/R‐ES were deposited onto the collection plate to form a thin film (Figure S1, Supporting Information). To evaluate the morphology of MSN, STFM/R, ES (unloaded electrospinning nanofibers) and 5F/STFM/R‐ES, transmission electron microscopy (TEM) and scanning electron microscopy (SEM) were applied. Representative images show the morphologies of MSN, STFM/R, ES and 5F/STFM/R‐ES (**Figure** **1**A,B). Dynamic Light Scattering (DLS) showed that the average size of STFM/R was ≈140 nm (Figure 1C). The average nanofiber diameter of 5F/STFM/R‐ES was ≈250 nm, which was measured by analyzing the TEM images of 5F/STFM/R‐ES (Figure 1D). After STFM/R and PLGA were stained with AF488 and AF647, respectively, the distribution of STFM/R in 5F/STFM/R‐ES was detected using laser scanning confocal microscopy (LSCM) (Figure 1E). The contact angle of 5F/STFM/R‐ES was 28°, indicating ideal hydrophilicity (Figure 1F,G). The results of the swelling experiments suggested that the degree of swelling of 5F/STFM/R‐ES was very low (Figure 1H). Using Inductively Coupled Plasma Mass Spectrometry (ICP‐MS), we detected the elemental contents of Fe and Mn in 5F/STFM/R‐ES, and the results suggested that STFM/R containing Fe and Mn was encapsulated in 5F/STFM/R‐ES (Figure 1I). The elemental mapping results from TEM‐EDX showed the elemental distribution in 5F/STFM/R‐ES (Figure 1J). The relatively low concentration of iron and manganese in the 5F/STFM/R‐ES complicates mapping detection. The spinning film's thickness and susceptibility to breakage under high voltage conditions hinder the acquisition of clear signals for elements present in low concentrations. Therefore, TEM mapping is employed solely as a qualitative method for detecting elements distribution, while ICP is utilized for precise quantification of metallic elements in this study. The loading efficiency of 5‐FU and R848 in 5F/STFM/R‐ES was measured by high‐performance liquid chromatography (HPLC). The results showed the loading efficiency of 5F and R848 in 5F/STFM/R‐ES were 0.47% and 0.48% (Figure S2, Supporting Information). The release of 5‐FU from 5F/STFM/R‐ES at different times and pH values were also measured using HPLC, and we observed that 5F/STFM/R‐ES could stably release 5‐FU in 20 days. The release of 5‐FU remained stable under both neutral and acidic conditions (Figure 1K, L). Furthermore, the release of iron, manganese, and R848 from 5F/STFM/R‐ES was evaluated. The concentrations of iron and manganese were quantified using ICP, while the concentration of R848 was determined via HPLC. The results indicated that the release of iron, manganese, and R848 was accelerated under conditions of low pH, attributed to the instability of TFM under acidic conditions (Figure S3, Supporting Information). This characteristic is advantageous for facilitating selective drug release in the acidic microenvironment of tumors. To examine the morphology of 5F/STFM/R‐ES following the release of 5‐FU and STFM/R, the 5F/STFM/R‐ES samples were immersed in phosphate‐buffered saline (PBS) at room temperature. After a period of twenty days, the morphology of the 5F/STFM/R‐ES was analyzed using scanning electron microscopy (SEM). (Figure 1M). Atomic force microscopy (AFM) revealed rough surfaces of 5F/STFM/R‐ES embedded with STFM/R nanoparticles (Figure 1N). These results indicated the successful synthesis of 5F/STFM/R‐ES.

### 2.2 5F/STFM/R‐ES Inhibited Melanoma Growth and Induced Ferroptosis In Vitro

**Figure 2:**
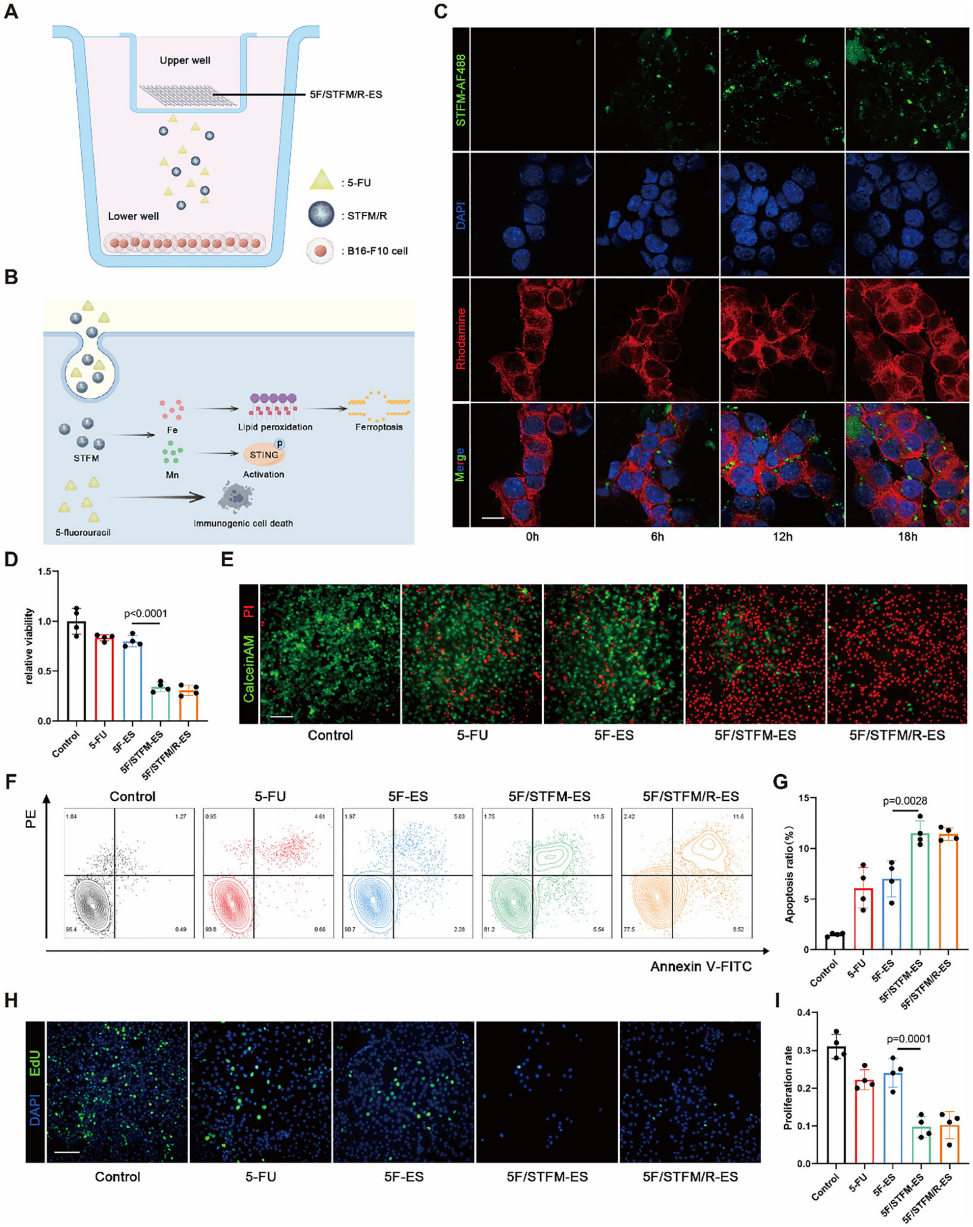
5F/STFM/R‐ES Inhibited Melanoma Growth and Induced Ferroptosis In vitro. A) Schematic showing the co‐incubation system for 5F/STFM/R‐ES and B16‐F10 cells in vitro. B) Schematic showing the antitumor effect of 5F/STFM. C) Images taken by LSCM showing the uptake of STFM/R in B16‐F10 at indicated times (scale bar equals 20 µm). STFMR were labeled with AF488. Rhodamine Phalloidin and DAPI were used to stain the cytoskeleton and nuclei of B16‐F10 cells (green for STFM/R, red for cytoskeleton and blue for nuclei). D) CCK‐8 assay revealing the cell viability of B16‐F10 treated with PBS, 5‐FU, 5F‐ES, 5F/STFM‐ES or 5F/STFM/R‐ES (n=4 experiments). E) Representative images of Live/Dead staining of B16‐F10 taken by fluorescence microscopy (green for live cells and red for dead cells) (scale bar equals 100 µm). F,G) Flow cytometry revealing the apoptosis ratio of B16‐F10 cell (n=4 experiments). H,I) Representative images and analysis of EDU assays showing the proliferating B16‐F10 cells in indicated groups (scale bar equals 100 µm) (n=4 experiments) (green for nuclei of proliferating cells and blue for nuclei of all cells). Data tested by parametric tests are presented as mean ± SEM. *p* values were determined by one‐way ANOVA with post hoc Bonferroni test.

**Figure 3:**
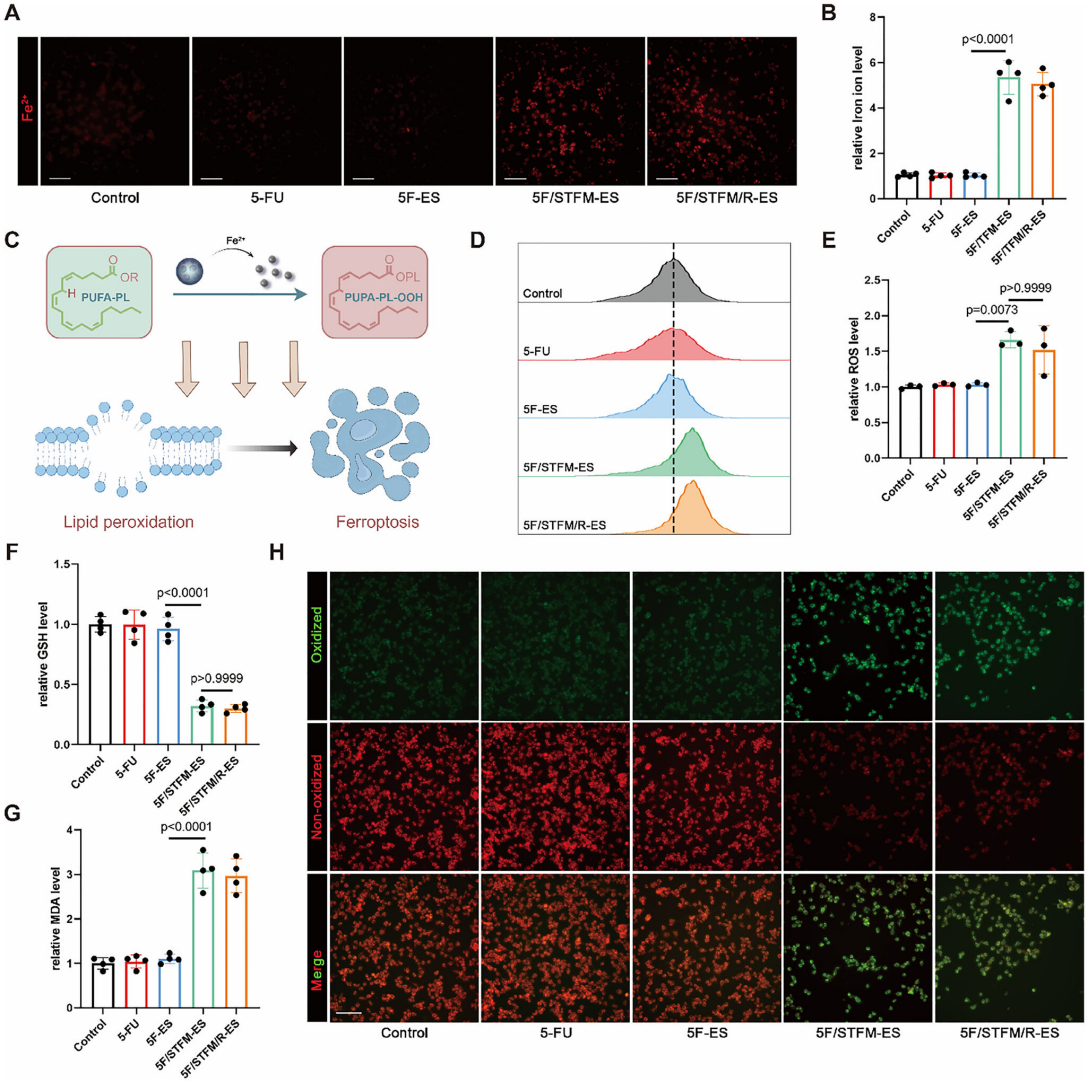
5F/STFM/R‐ES Induced Ferroptosis In vitro. A,B) Analysis and representative images showing the relative concentration of Fe^2+^ in B16‐F10 by using RhoNox‐1 probe (red for Fe^2+^) (scale bar equals 100 µm) (n=4 experiments). C) Schematic showing the mechanism of ferroptosis induced by 5F/STFM/R‐ES. The Fe^2+^ released from 5F/STFM/R‐ES initiate lipid peroxidation, subsequently resulting in cell membrane rupture, ferroptosis, and cell death. D,E) ROS level in B16‐F10 was measured by DCFH‐DA (n=3 experiments). F) GSH levels of B16‐F10 in indicated groups (n=4 experiments). G) MDA assay revealed the MDA levels in B16‐F10 cells (n=4 experiments). H) C11 BODYPI 581/591 staining was employed to measure the lipid peroxidation level of B16‐10 cells (red for non‐oxidized lipid and green for oxidized lipid) (scale bar equals 100 µm). Data tested by parametric tests are presented as mean ± SEM. *p* values were determined by one‐way ANOVA with post hoc Bonferroni test.

To investigate the anti‐tumor effect of 5F/STFM/R‐ES in vitro, B16‐F10 cells were incubated with 5F/STFM/R‐ES in the incubation system as indicated (**Figure** **2**A). 5F/STFM/R‐ES was placed on the upper well while B16‐F10 were cultured in the lower well. The micropores with an 8 µm pore size at the base of the upper well allow the passage of 5‐FU and STFM/R released by 5F/STFM/R‐ES into the lower well. Upon release from the 5F/STFM/R‐ES, STFM/R and 5‐FU were subsequently internalized by the B16‐F10 cells, leading to the induction of ferroptosis, activation of the STING pathway, and ICD (Figure 2B). For the assessment of STFM/R release and uptake in melanoma cells, STFM/R was labeled with AF488 prior to its incorporation into 5F/STFM/R‐ES. Subsequently, the B16‐F10 cells were exposed to 5F/STFM/R‐ES, and observed using LSCM at designated time points. STFM/R‐AF488 gradually accumulated in the melanoma cells, indicating the effective release of STFM/R from 5F/STFM/R‐ES (Figure 2C). The antitumor effect of 5F/STFM/R‐ES was evaluated using CCK‐8 and live/dead assays (Figure 2D,E). 5F/STFM/R‐ES significantly decreased the viability of the melanoma cells and increased the percentage of dead cells. There was no significant difference in the viability of melanoma cells treated with low concentrations of 5‐FU or 5‐FU loaded electrospinning nanofibers (5F‐ES). When STFM or STFM/R was loaded into ES, the viability of melanoma cells was dramatically inhibited and the dead cell ratio also increased, but there was no remarkable difference between the 5F/STFM ‐ES and 5F/STFM/R‐ES groups. These results suggest that STFM plays a key role in killing melanoma cells. The apoptotic level of melanoma cells was detected using flow cytometry. Apoptosis assays revealed that the apoptosis level of melanoma cells treated with 5F/STFM/R‐ES or 5F/STFM‐ES was the highest, which was consistent with the results of the CCK‐8 and live/dead assays (Figure 2F,G). The proliferation of melanoma cells was measured using EdU assays, and the results indicated that the number of positive cells was significantly decreased in the 5F/STFM/R‐ES group (Figure 2 H,I). After being absorbed by melanoma cells, Fe^2+^ could be released from STFM/R. Therefore, we first detected Fe^2+^ in melanoma using the Fe^2+^ probe RhoNox‐1, and the results indicated that STFM/R upregulated Fe^2+^ in melanoma (**Figure** **3**A,B). Fe^2+^ could participate Fenton reaction, consumes GSH, and generates a large amount of ROS, which finally induces lipid peroxidation and ferroptosis (Figure 3C).^[^ ^40^ ^]^ ROS and GSH levels in melanoma cells were further assessed, and TFM significantly increased ROS levels and decreased GSH levels in melanoma (Figure 3D–F). Malondialdehyde (MDA) is the main product of lipid peroxidation; therefore, we also measured MDA levels in melanoma cells.^[^ ^41^ ^]^ MDA assays revealed that melanomas in the 5F/STFM/R‐ES and 5F/STFM‐ES groups had higher MDA levels than the other groups (Figure 3G). Furthermore, the lipid peroxidation level of melanoma was measured using the BODIPY 581/591 C11 probe, and the lipid peroxidation level of melanoma in 5F/STFM/R‐ES and 5F/STFM‐ES cells was markedly increased (Figure 3H). Furthermore, the capacity of STFM and 5F/STFM/R‐ES to induce ferroptosis was evaluated in comparison to the positive control group (0.5 µg mL^−1^ RSL3 or 5 µg mL^−1^ Erastin). The results demonstrated that both STFM and 5F/STFM/R‐ES effectively induced ferroptosis in B16‐F10 cells (Figure S4, Supporting Information). These results demonstrate that 5F/STFM/R‐ES effectively induced ferroptosis in melanoma and inhibited melanoma progression in vitro.

### 2.3 5F/TFM/R‐ES Activated Antitumor Immune Response In Vitro

**Figure 4:**
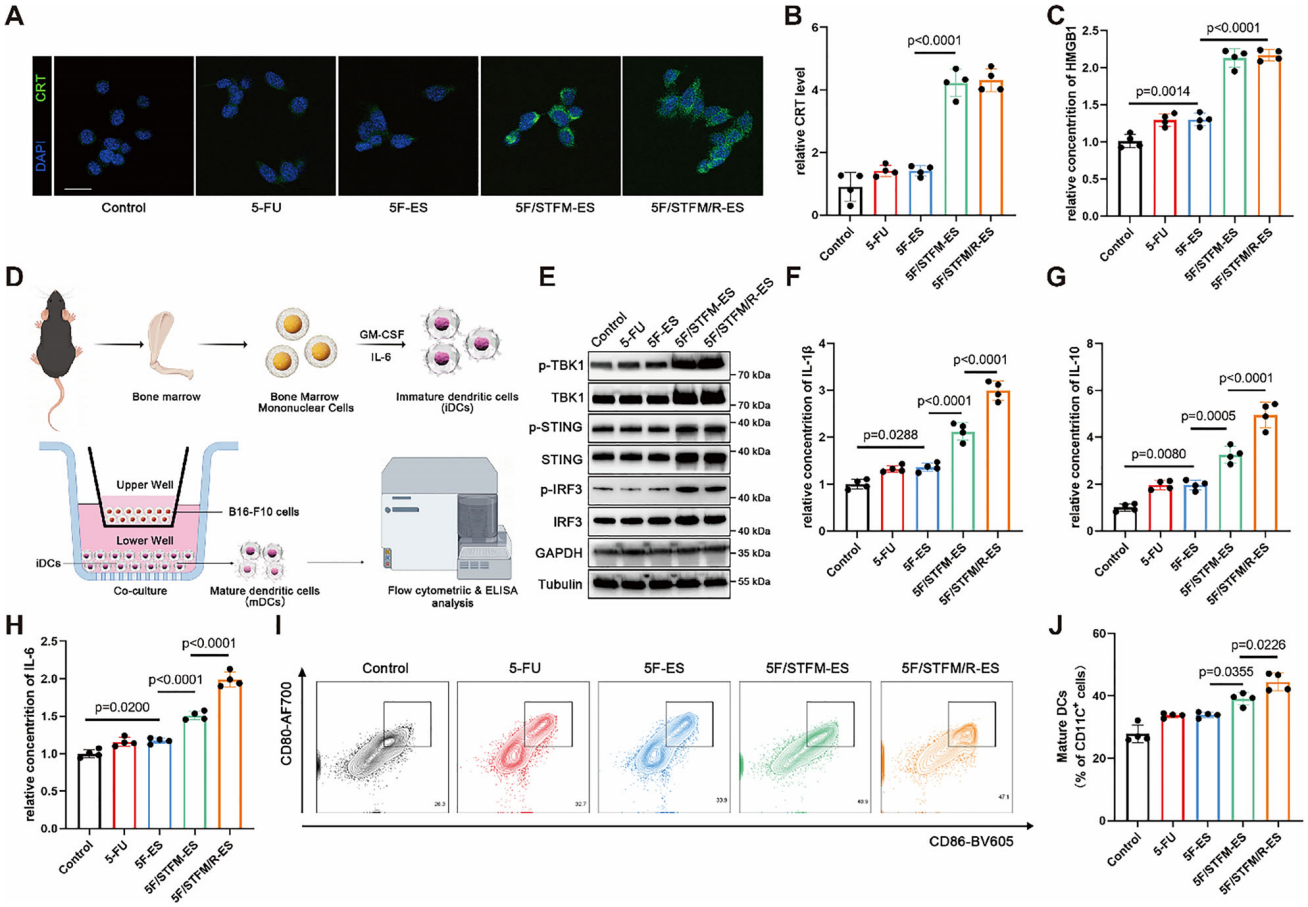
5F/TFM/R‐ES activated antitumor immune response in vitro. A,B) Representative immunofluorescence images and analysis (mean fluorescence intensity) showing the expression of CRT in B16‐F10 cells (green for CRT and blue for nuclei) (n = 4 experiments; scale bar equals 20 µm). C) ELISA results showing the release level of HMGB1 by B16‐F10 cells (n=4 experiments). D) Schematic illustrating the process of obtaining BMDCs and the co‐culture system of BMDCs and B16‐F10 cells. E) Western blots revealed the expression of STING, p‐STING, TBK1, p‐TBK1, IRF3, and p‐IRF3 expression in BMDCs. F–H) ELISA results showing the release level of IL‐10, IL‐1β, and IL‐6 by BMDCs (n=4 experiments). I,J) Flow cytometry showing the ratio of mature DCs (CD80^+^ and CD86^+^) in various groups (n=4 experiments). Data tested by parametric tests are presented as mean ± SEM. *p* values were determined by one‐way ANOVA with post hoc Bonferroni test.

ICD is a type of cell death that involves activation of the antitumor immune response.^[^ ^42^ ^]^ The release of damage‐associated molecular patterns (DAMPs) from dying tumor cells can activate tumor‐specific immune responses.^[^ ^43^ ^]^ And DAMPs include high‐mobility group box‐1 (HMGB1), calreticulin (CRT), extracellular release of adenosine triphosphate (ATP), and heat shock proteins (HSP70 and HSP90).^[^ ^44^ ^]^ In the drug delivery system of 5F/STFM/R‐ES, both 5‐FU and ferroptosis are recognized as ICD inducers, and Mn^2+^ could effectively initiate an antitumor immune response by activating the cGAS‐STING signaling pathway; thus, we performed in vitro experiments to investigate the function of 5F/STFM/R‐ES in immune activation. We first detected the expression of CRT and the release of HMGB1, the main DAMPs, using immunofluorescence and ELIAS assays. Immunofluorescence analysis revealed that CRT expression was upregulated after treatment with 5F/STFM/R‐ES or 5F/STFM‐ES (**Figure** **4**A,B). The ELISA results indicated that 5F/STFM/R‐ES or 5F/STFM‐ES increased the release of HMGB1 (Figure 4C). Afterwards, bone marrow‐derived dendritic cell (BMDCs) was separated from mice and co‐cultured with melanoma that pretreated with indicated drugs (Figure 4D). Protein expression in BMDCs was measured by western blotting, flow cytometry, and ELISA. The results of western blotting suggested that the expression of key regulators in the cGAS‐STING signaling pathway, including p‐STING, p‐TBK1, and pIRF3, was upregulated after melanoma treatment with 5F/STFM/R‐ES or 5F/STFM‐ES, indicating that the antitumor immune response of BMDCs targeting melanoma was activated (Figure 4E). By employing ELISA assays, the concentration of IL‐1β, IL‐6, and IL‐10 were measured, and the results demonstrated 5F/STFM/R‐ES or 5F/STFM‐ES effectively increased the expression of these cytokines (Figure 4F–H). The expression of CD80 and CD86, which are markers of BMDCs activation, was detected by flow cytometry. The results showed that the ratio of CD80 and CD86 positive BMDCs was significantly increased in the 5F/STFM/R‐ES and 5F/STFM‐ES groups (Figure 4I,J). These results show that 5F/STFM/R‐ES efficiently enhanced the function of BMDCs and stimulated an antitumor immune response.

### 2.4 5F/TFM/R‐ES Inhibited Melanoma Growth In Vivo

**Figure 5:**
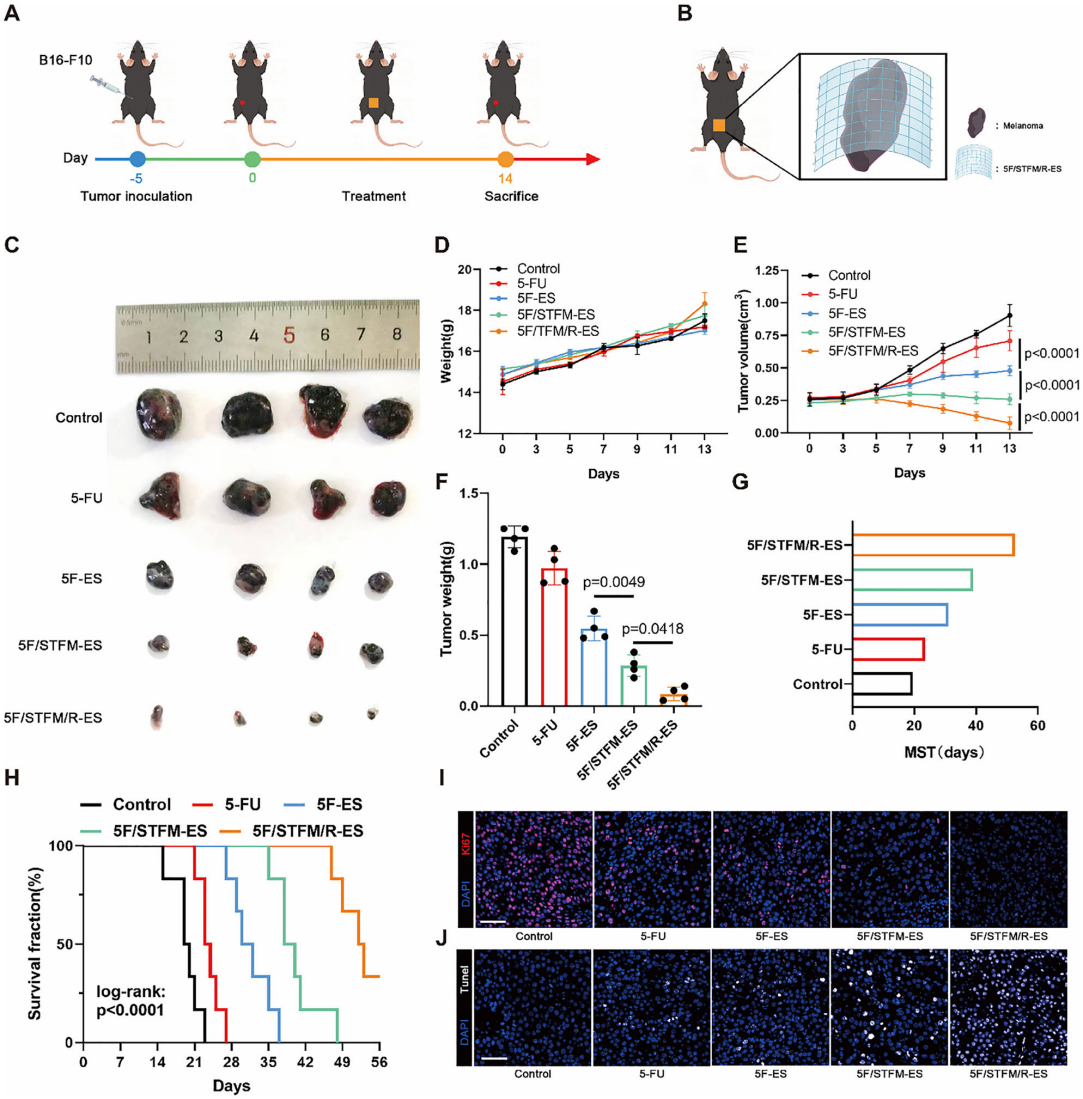
5F/STFM/R‐ES inhibited melanoma growth in vivo. A,B) Schematic illustrating the establishment and treatment of melanoma mice models. C) Melanomas of the mice with indicated treatment. D) The body weight changes during different treatments (n=4 mice). E) Tumor volume changes in each group with different treatments (n=4 mice). F) Tumor weight in each group with different treatments (n=4 mice). G,H) Survival analysis of the mice in various groups, and the median survival time was measured (n=6 mice). I,J) Representative images of TUNEL staining and immunofluorescence staining for Ki67 (scale bar equals 100 µm). Data tested by parametric tests are presented as mean ± SEM. *p* values were determined by two‐way ANOVA with post hoc Bonferroni test in D and E, one‐way ANOVA with post hoc Bonferroni test in F and log rank in H.

To further investigate the antitumor effect in vivo, we constructed transplanted melanoma models. Five days after the inoculation, the mice were randomly divided into five groups. In the control and 5‐FU groups, mice underwent a sham operation and tail vein injections of physiological saline or 5‐FU. In the 5F‐ES, 5F/STFM‐ES, and 5F/STFM/R‐ES groups, the mice were injected with physiological saline, and the indicated nanofibers membranes were surgically placed on the tumor surface (**Figure** **5**A,B). The image of the melanomas after treatment indicated 5F/STFM/R‐ES significantly suppressed melanoma progression in vivo (Figure 5C). Body weight and tumor growth were monitored every 2 days. The body weights of mice from the different groups showed no significant differences (Figure 5D). The tumor volume in the 5‐FU group was larger than that in the 5F‐ES group, indicating that the treatment efficiency of local administration via nanofibers was much higher than that of intravenous administration. The tumor volume in the 5F/STFM‐ES group was lower than that in the 5F‐ES group, and the tumor volume in the 5F/STFM/R‐ES group decreased further (Figure 5D; Figure S5, Supporting Information). Tumor weight analysis showed the same conclusion (Figure 5F). Analysis of the survival fraction and median survival time of the mice in the various groups showed that 5F/STFM‐ES effectively prolonged the survival time of the mice, and 5F/STFM/R‐ES further increased this effect (Figure 5G,H). To assess apoptosis and proliferation levels in melanoma, TUNEL staining and immunofluorescence staining for Ki67 were conducted (Figure 5I,J). The results indicated that 5F/STFM/R‐ES treatment induced apoptosis and inhibited the growth of melanoma. These results suggest that local administration via nanofibers is a more effective method, and that 5F/STFM/R‐ES could significantly inhibit melanoma growth in vivo.

### 2.5 5F/STFM/R‐ES Activated Antitumor Immune Response In Vivo

**Figure 6:**
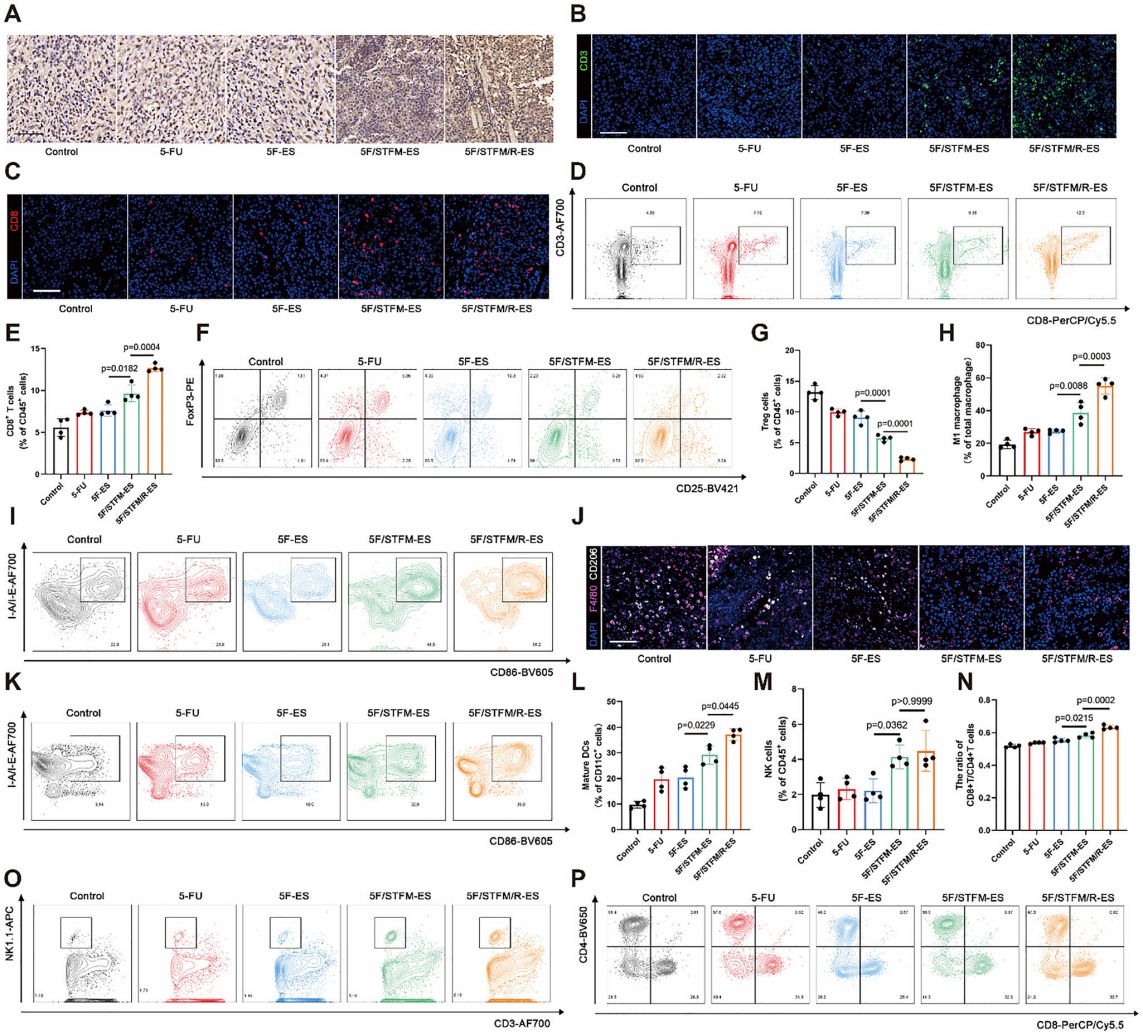
5F/STFM/R‐ES activated antitumor immune response in vivo. A) Representative immunohistochemistry images showing the expression of HMGB1 in melanoma tissues (scale bar equals 100 µm). B) Representative immunofluorescence images showing the CD3^+^ T cells in melanoma tissues (green for CD3 and blue for nuclei) (scale bar equals 100 µm). C) Representative immunofluorescence images showing the CD8^+^ T cells in melanoma tissue (red for CD8 and blue for nuclei) (scale bar equals 100 µm). D,E) Flow cytometry revealed the ratio of CD8^+^ T cells in CD45^+^ cells in melanoma tissues (n=4 mice). F,G) Flow cytometry revealed the ratio of CD25^+^/FoxP3^+^ Tregs in CD45^+^ cells in melanoma tissues (n=4 mice). H,I) Flow cytometry revealed the ratio of CD86^+^/I‐A/I‐E^+^ M1 macrophages in CD11b^+^/F4/80^+^ macrophage cells in melanoma tissues (n=4 mice). J) Representative immunofluorescence images showing the expression of F4/80 and CD206 in melanoma tissues (white for CD206, purple for F4/80 and blue for nuclei) (scale bar equals 100 µm). K,L) Flow cytometry revealed the ratio of CD86^+^/I‐A/I‐E^+^ mDCs in CD11c^+^ cells in melanoma tissues (n=4 mice). M,O) Flow cytometry revealed the ratio of CD3‐/NK1.1^+^ NK cells in CD45^+^ cells in melanoma tissues (n=4 mice). N,P) Flow cytometry revealed the ratio of CD4^+^T cell to CD8^+^T cells in spleen (n=4 mice). Data tested by parametric tests are presented as mean ± SEM. *p* values were determined by one‐way ANOVA with post hoc Bonferroni test.

To evaluate the immunoregulatory effects of 5F/STFM/R‐ES in vivo, we detected changes in the immune microenvironment of melanoma tissues. The expression of HMGB1 in melanoma tissues was measured by immunohistochemistry, and the results indicated that HMGB1 was transferred from the nucleus to the cytoplasm and extracellular matrix (**Figure** **6**A). CD3^+^ and CD8^+^T cells were stained with immunofluorescence (Figure 6B,C). The quantity of CD3+ T cells and CD8+ T cells was elevated in both the 5F/STFM‐ES and 5F/STFM/R‐ES groups, with the 5F/STFM/R‐ES‐treated melanoma exhibiting the highest levels. The ratio of CD3^+^/CD8^+^ T cells to CD45^+^ cells was measured by flow cytometry (Figure 6D,E). These results indicates that 5F/STFM/R‐ES increases the number of CD8+T cells in melanoma tissues. In addition, the number of Tregs in melanoma tissues was also decreased (Figure 6F,G). To verify the function of R848 in 5F/STFM/R‐ES, we measured the ratio of M1 macrophages in the melanoma tissues. Flow cytometry and immunofluorescence analyses suggested that the ratio of M1 macrophages to total macrophages in the 5F/STFM/R‐ES group was the highest (Figure 6H–J). To comprehensively detect changes in the immune microenvironment within melanomas, we determined the number of mDCs and NK cells by flow cytometry, and the results suggested 5F/STFM/R‐ES increased the number of mDCs and NK cells in melanoma tissues (Figure 6K–M,O). Additionally, we assessed the ratio of CD8^+^T/CD4^+^ T cells in the spleen. Flow cytometry showed that the ratio of CD8^+^T/CD4^+^ T cells in the spleens of the 5F/STFM/R‐ES group increased (Figure 6N,P). Subsequently, ELISA assays were performed to assess the expression of the key cytokines, which showed that the expression of IL‐1β, IL‐6, IL10, and IFN‐γ, were all upregulated after treated with 5F/STFM/R‐ES (Figures S6–S9, Supporting Information). 5F and Fe^2+^ can induce ICD in tumor tissues, Mn^2+^ can initiate the immune response by upregulating the cGAS‐STING signaling pathway, and R848 can further enhance the polarization of macrophages from M2 to M1. These results demonstrate that 5F/STFM/R‐ES effectively activated the antitumor immune response in melanoma.

### 2.6 5F/TFM/R‐ES Inhibited Melanoma Metastasis and Recurrence In Vivo

**Figure 7:**
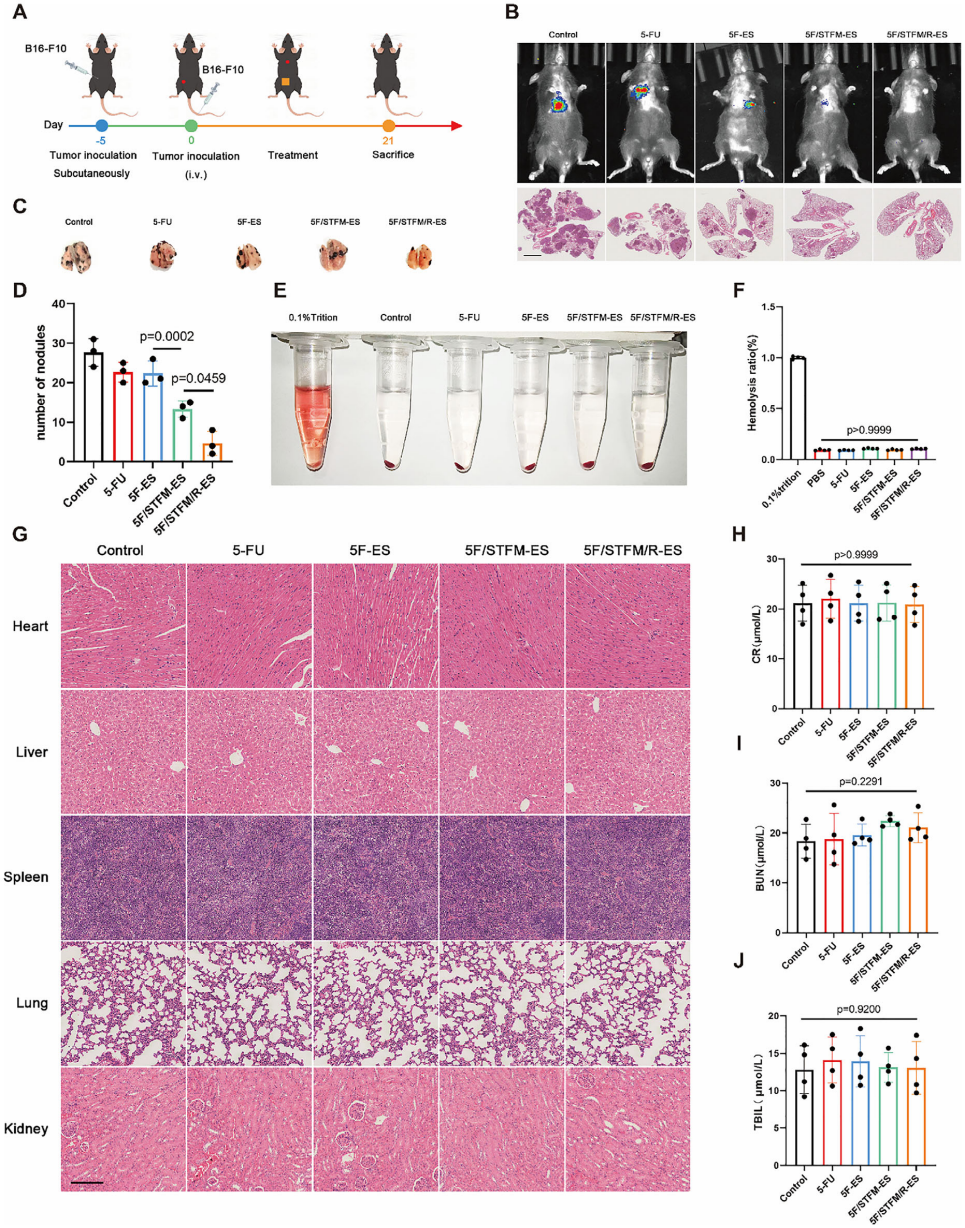
5F/TFM/R‐ES inhibited melanoma metastasis and biosafety. A) Schematic illustrating the establishment and treatment of melanoma metastasis models. B) Representative images taken by small animal live imaging systems and HE staining showing the metastasis of melanoma on the lungs (scale bar equals 2 mm). C) Representative images showing the lungs of the mice in each group. D) Analysis of the number of nodules on the lungs (n = 3 mice). E,F) Hemolysis assays revealing the hemolysis ratio after treated with indicated drugs (n = 4 experiments). G) HE staining showing the structure of the main organs (scale bar equals 100 µm). H–J) Serum biochemical indicators analysis of creatinine (CR), blood urea nitrogen (BUN), total bilirubin (TBIL) (n=4 mice). Data tested by parametric tests are presented as mean ± SEM. *p* values were determined by one‐way ANOVA with post hoc Bonferroni test.

To further investigate the role of 5F/STFM/R‐ES in the metastasis and recurrence of melanoma, we constructed mouse models of metastasis and recurrence of melanoma as indicated (**Figure** **7**A; Figure S2, Supporting Information). Melanoma metastasis was monitored in small animals by using live imaging systems. The fluorescence of melanoma in the lungs of the control group was significantly stronger than that in the 5F/STFM‐ES and 5F/STFM/R‐ES groups (Figure 7B). The number of nodules in the lungs was also analyzed, and the results showed that the number of nodules in the 5F/STFM‐ES group was the lowest (Figure 7C,D). The images of the and lung HE staining results also confirmed this conclusion (Figure 7B). In the recurrence model, the melanomas were removed with 2 mm × 2 mm × 2 mm of tumor tissue left for further treatment (Figure S10, Supporting Information). The results suggested that 5FSTFM/R‐ES effectively suppressed melanoma recurrence (Figures S11–S13, Supporting Information). To further evaluate the biosafety of 5F/STFM/R‐ES, a hemolysis test was performed, which indicated that 5F‐ES, 5F/STFM‐ES, and 5F/STFM/R‐ES did not enhance the hemolysis ratio (Figure 7E,F). HE staining results of the hearts, lungs, kidneys, spleens, and livers of the different groups were not significantly different (Figure 7G). The levels of alanine aminotransferase (ALT), creatinine (CR), blood urea nitrogen (BUN), total bilirubin (TBIL), and creatine kinase (CK) were measured, and the results showed no significant differences between the groups (Figure 7H–J; Figure S14, Supporting Information). These results demonstrate that 5F/STFM/R‐ES is biosafe and can inhibit melanoma metastasis and recurrence.

## 3 Conclusion

In conclusion, we developed a nanofiber‐based drug delivery system that significantly inhibited melanoma progression. In this system, the application of electrostatic spinning and biodegradable nanofibers provided a platform for high drug loading and sustained release, which reduced systemic side effects and ensured prolonged therapeutic exposure at the tumor site. Furthermore, we loaded 5‐fluorouracil into the nanofibers to directly kill melanoma cells and induce ICD to improve the immune response. We then constructed a metal‐polyphenol network TFM with Fe^2+^, Mn^2+^ and tannic acid. TFM efficiently induces ferroptosis in melanoma cells by releasing Fe^2+^. Ferroptosis in melanoma on the hand directly inhibited melanoma progression and on the other hand triggered ICD in melanoma and stimulated the immune response. Subsequently, Mn^2+^ released from the TFM could be further released into the tumor microenvironment after cell death, which could be taken up by iDCs and upregulate the cGAS‐STING signaling pathway in iDCs. The cGAS‐STING signaling pathway enhances the downstream expression of TBK1 and IRF3, which promotes antigen uptake and maturation of iDCs. To ensure stable delivery of TFM into melanoma cells, TFM was coated on the surface of MSN and further loaded into nanofibers. R848 was encapsulated in the core of MSN to regulate macrophage polarization in the tumor microenvironment.^[^ ^37^ ^]^ By this way, 5F/STFM/R‐ES could activate and enhance the antitumor immune response. 5F/STFM/R‐ES significantly inhibited melanoma growth in vitro and in vivo. In melanoma recurrence and metastasis models, 5F/STFM/R‐ES suppressed recurrence and metastasis. The nanofiber system 5F/STFM/R‐ES not only enhances localized drug action but also minimizes damage to the surrounding healthy tissues, providing a more precise and effective treatment strategy than conventional chemotherapy. A critical advantage of this system is its ability to induce multiple cell death pathways, including chemotherapy‐induced apoptosis, Fe^2+^‐induced ferroptosis, and immunotherapy‐induced cell death. Enhancing cell death through multiple induction pathways increases the overall success of the treatment, presenting a more comprehensive approach to tumor cell eradication. Comprehensive in vitro and in vivo assessments highlighted the effectiveness of this approach, demonstrating its potential for melanoma therapy. While certain challenges remain, such as the unsuitability of some drugs for electrospinning under high voltage, which constrains the selection of applicable drugs, our findings pave the way for novel applications of metal‐polyphenol networks and electrospinning in inducing ferroptosis and activating the immune response. This suggests a substantial potential for this technology in the field of oncology.

## 4 Experimental Section

### Synthesis of STFM/R

To fabricate the S/R, 12.5 mg of R848 and 10 mg of MSN were dispersed in 3 mL of DMSO. After stirring overnight, the S/R was collected by centrifugation and washed three times with water. To synthesize STFM/R, 23 mg of FeCl_2_ and MnCl_2_, 80 mg of polyvinylpyrrolidone (PVP), and 58 mg of S/R were dissolved in 4 mL of ultrapure water and stirred for 5 min. Tannic acid (10 mg mL^−1^) was then added to the solution and stirred for 5 min. STFM/R was obtained by centrifugation and washed three times with water.

### Electrospinning

To fabricate 5F/STFM/R‐ES, 350 mg PLGA, 2 mg 5‐FU and 75.53 mg STFM/R were dissolved in 2.5 mL trifluoroethanol and stirred at room temperature overnight. The obtained homogeneous solution was then loaded into the needle of a syringe using a programmable pump at a speed of 0.0025 mL h^−1^ with a 22‐G blunt metal needle. A voltage of 15 KV was applied between the needle and the rotating collector, which were 15 cm apart. To remove the remaining organic solvents, the gathered electrospun fibers were vacuum‐freeze‐dried overnight.

### Characterization of STFM/R and 5F/STFM/R‐ES

The morphologies of STFM/R and 5F/STFM/R‐ES were measured using transmission electron microscopy (TEM; JEOL JEM‐1400, Japan) and scanning electron microscopy (SEM; Carl Zeiss G300, Germany). Transmission electron microscope elemental mapping was performed using Thermo Fisher Talos SuperX. The zeta potentials and hydrodynamic diameters of the STFM/R were measured using a Malvern Zetasizer. The diameter of 5F/STFM/R‐ES were measured by analyzing the TEM images of 5F/STFM/R‐ES with ImageJ software. The contact angle measuring instrument (DSA100, Kruss) was used to characterize the wettability of the 5F/STFM/R‐ES. Atomic force microscopy (AFM, Bioscope Resolve, Bruker) was employed to obtain topography images of the 5F/STFM/R‐ES.

### Cell Culture

Murine melanoma B16‐F10 cells (RRID: CVCL_0159) were purchased from ProCell Life Science and Technology Co. Ltd. (Hubei, China). The B16‐F10 cell line was contamination‐free, and authentication of B16‐F10 cells was conducted via short tandem repeat (STR) profiling at Procell Life Science & Technology Co., Ltd. B16‐F10 cells was cultured in Roswell Park Memorial Institute (RMPI) 1640 with 10% fetal bovine serum (FBS) in a humidified atmosphere at 37 °C with 5% CO_2_.

### Cellular Uptake and In Vitro Assays

To assess the uptake of STFM/R by melanoma cells in vitro, STFM/R was incubated with AF488 for 12 h at room temperature before incorporated into 5F‐STFM/R‐ES. The B16‐F10 cells were seeded in 24‐well plates (5 × 10^4^ cells per well) and cultured at 37 °C overnight. Next, 5F/STFM/R‐ES was introduced to the upper well of the co‐incubation system and incubated for specified durations. After incubation, the cells were washed with phosphate buffered saline (PBS) and fixed with 4% paraformaldehyde. Rhodamine Phalloidin and DAPI were used to stain the cytoskeleton and nuclei of B16‐F10 cells. Afterwards, the cells were visualized using a fluorescence microscope (Leica, Germany).

In vitro experiments used five groups: Control, 5‐FU, 5F‐ES, 5F/STFM‐ES, and 5F/STFM/R‐ES. The dosage of 5‐FU and R848 were quantified by HPLC, and the dosage of TFM was quantified by ICP. Final concentrations were 4.7 µg mL^−1^ 5‐FU in culture medium (in 5‐FU, 5F‐ES, 5F/STFM‐ES and 5F/STFM/R‐ES group), 19.9 µg mL^−1^ Fe (in 5F/STFM‐ES and 5F/STFM/R‐ES group) and 4.8 µg mL^−1^ R848 (in 5F/STFM/R‐ES group). The cells in the 5‐FU group or the control group were treated with 5‐FU or PBS respectively, ensuring that the volumes of 5‐FU and PBS were equivalent. The cells in 5F‐ES, 5F/STFM‐ES, and 5F/STFM/R‐ES group were administered within the co‐incubation system. (Figure 2A).

### Cell Viability and Proliferation Assays

The viability of B16‐F10 cells was measured using the CCK‐8 assay (Biosharp, China) following the manufacturer's instructions. In summary, the B16‐F10 cells were seeded in 24‐well plates (5 × 10^4^ cells per well) and incubated at 37 °C overnight. Subsequently, PBS, 5‐FU, 5F‐ES, 5F/STFM‐ES, and 5F/STFM/R‐ES were administered into the respective groups as previously described. Final concentrations were 4.7 µg mL^−1^ 5‐FU in culture medium (in 5‐FU, 5F‐ES, 5F/STFM‐ES, and 5F/STFM/R‐ES group), 19.9 µg mL^−1^ Fe (in 5F/STFM‐ES and 5F/STFM/R‐ES group) and 4.8 µg mL^−1^ R848 (in 5F/STFM/R‐ES group). After 24 to 48 h, the culture medium was replaced with 1640 medium supplemented with 10% CCK‐8 reagent. Following a 1‐hour incubation period, the absorbance at 450 nm for each well was measured using a microplate reader. The proliferation of B16‐F10 cells was monitored using ethynyl‐2‐deoxyuridine (EdU) assays (RiboBio, China) following the manufacturer's instructions. Briefly, 20 µm EdU was added to B16‐F10, and incubated for 2 h. The cells were fixed in 4% paraformaldehyde. After washing with PBS and perforation with 5% triton, the cells were stained with Hoechst 33342 and Apollo 488. Images were captured using LSCM, and the ratio of positive cells was measured using Image J software (National Institutes of Health, Bethesda, MD).

### Western Blot

Melanoma cells in various groups were lysed in RIPA (Thermo, USA) supplemented with a phosphatase and protease inhibitors (Sigma, USA). The protein concentration was measured with BCA protein assay kit (Beyotime, China). Protein lysates were separated on SDS polyacrylamide gels (10% SDS‐PAGE) and transferred to a polyvinyl difluoride (PVDF) membrane (Merck Millipore, USA). After blocked with 5% defatted milk for 1 h, the membranes were incubated with indicated primary antibodies and horseradish peroxidase (HRP)‐conjugated secondary antibody. The blots were visualized using Immobilon Western Chemiluminescent HRP Substrate (Sigma‐Aldrich, St Louis, MO, USA). The following antibodies were used for western blots: HRP‐conjugated Affinipure goat anti‐Rabbit IgG(H+L) (Proteintech, China); HRP‐conjugated Affinipure goat anti‐Mouse IgG(H+L) (Proteintech, China); anti‐alpha tubulin (Proteintech, China); anti‐STING (Proteintech, China); anti‐pSTING (abclonal, China); anti‐TBK1 (Proteintech, China); anti‐pTBK1 (abcam, USA); anti‐IRF3 (Proteintech, China); anti‐pIRF3 (Proteintech, China); anti‐GAPDH (Proteintech, China).

### Live/Dead Cell Viability Assay

The B16‐F10 cells were seeded in 24‐well plates (5 × 10^4^ cells per well) and cultured at 37 °C overnight. Subsequently, PBS, 5‐FU, 5F‐ES, 5F/STFM‐ES, and 5F/STFM/R‐ES were administered into the respective groups as previously described. Final concentrations were 4.7 µg mL^−1^ 5‐FU in culture medium (in 5‐FU, 5F‐ES, 5F/STFM‐ES, and 5F/STFM/R‐ES group), 19.9 µg mL^−1^ Fe (in 5F/STFM‐ES and 5F/STFM/R‐ES group) and 4.8 µg mL^−1^ R848 (in 5F/STFM/R‐ES group). After 24 to 48 h, the cells were then stained with a Live/Dead Cell Staining Kit (Beyotime, China) according to the manufacturer's instructions. The results were recorded using a fluorescence microscope (Leica, Germany).

### Apoptosis Detection In Vitro

The B16‐F10 cells were seeded in 24‐well plates (5 × 10^4^ cells per well) and cultured at 37 °C overnight. Subsequently, PBS, 5‐FU, 5F‐ES, 5F/STFM‐ES, and 5F/STFM/R‐ES were administered into the respective groups as previously described. Final concentrations were 4.7 µg mL^−1^ 5‐FU in culture medium (in 5‐FU, 5F‐ES, 5F/STFM‐ES, and 5F/STFM/R‐ES group), 19.9 µg mL^−1^ Fe (in 5F/STFM‐ES and 5F/STFM/R‐ES group) and 4.8 µg mL^−1^ R848 (in 5F/STFM/R‐ES group). After an incubation period of 24 to 48 h, the cells in each group were collected and stained using Annexin V‐FITC Apoptosis Detection Kit (Beyotime, China) following the manufacturer's protocol. The ratio of apoptosis was measured by flow cytometry with indicated gating strategy (Figure S15, Supporting Information).

### Establishment and Treatment of Murine Melanoma Models

C57BL/6J mice (6‐week‐old female) were purchased from the Beijing Vital River Laboratory Animal Technology (Beijing, China). To construct the orthotopic models, B16‐F10 cells (1 × 10^6^) were resuspended in PBS and subcutaneously injected into mice. Five days later, the mice were randomly divided into five groups and received the indicated treatments. After 14 days of treatment, the mice were sacrificed. To establish a pulmonary metastasis model of melanoma in mice, B16‐F10 cells (1 × 10^6^) were resuspended in PBS and subcutaneously injected into the mice. Five days later, B16‐F10 cells (3 × 10^5^) were injected into the tail vein. Afterwards, the mice were randomly divided into five groups and subjected to the indicated treatments. Twenty‐one days later, the mice were euthanized. Melanoma progression in the lungs of mice was monitored using live imaging systems. To construct the melanoma recurrence model, B16‐F10 cells (1 × 10^6^) were resuspended in PBS and subcutaneously injected into mice. Five days later, the melanomas were surgically removed, leaving 2 mm × 2 mm × 2 mm of melanoma tissue. Mice were randomly divided into five groups and received the indicated treatments. After 14 d, the mice were sacrificed.

The treatment methods in animal experiments are as follows. Control group: tail vein injection of 100 µL normal saline; 5‐FU group: tail vein injection of 5‐FU; 5F‐ES, 5F/STFM‐ES, and 5F/STFM/R‐ES group: the nanofibrous membranes were surgically placed on the tumor surface. Final concentrations in animal experiments were 2.5 mg kg^−1^ 5‐FU (in 5‐FU, 5F‐ES, 5F/STFM‐ES, and 5F/STFM/R‐ES group), 10.6 mg kg^−1^ Fe (in 5F/STFM‐ES and 5F/STFM/R‐ES group), and 2.6 mg kg^−1^ R848 (in 5F/STFM/R‐ES group).

### In Vivo Flow Cytometry

In vivo flow cytometry was performed to analyze immune cells in melanoma tissues. Melanoma tissue was collected and cut into small pieces. The tissues were digested in RMPI 1640 medium with Collagenase IV (1 mg mL^−1^; Solarbio) and Deoxyribonuclease I (0.2 mg mL^−1^, Solarbio). After digestion, single‐cell suspensions were incubated with anti‐CD16/32 antibody (BioLegend, USA) to block Fc. Afterwards, dead cell staining kit (Zombie NIR Fixable Viability Kit, Bioleged) was applied to mark the dead cells. The cells were then stained with fluorophore‐labelled antibodies against CD45, CD11c, CD11b, F4/80, CD206, CD86, I‐A/I‐E, CD83, CD80, CD3, CD4, CD8, Foxp3 and NK1.1 (Bioleged, USA) for 30 min. As for the staining of CD206, the cells were fixed and permeabilized before staining with Cyto‐Fast Fix/Perm Buffer Set (Bioleged, USA). After staining, the cells were analyzed using flow cytometry (Cytek Aurora, USA).

### Immunofluorescence and Immunohistochemistry

Immunofluorescence staining for melanoma cells was performed to detect CRT expression. B16‐F10 cells were fixed in 4% paraformaldehyde and stained with an anti‐CRT antibody (Proteintech). The melanoma tissues were fixed in 4% paraformaldehyde and embedded in paraffin. Hematoxylin and eosin (H&E) staining revealed the progression of melanoma in the lungs and the structures of the main organs, including the lungs, kidneys, heart, spleen, and liver. Immunohistochemistry staining revealed the expression of HMGB1 in melanoma tissues with anti‐HMGB1 (Proteintech, China). Immunofluorescence staining of melanoma tissues was conducted to assess the expression of CD3, F4/80, and CD206 using anti‐CD3, anti‐F4/80, and anti‐CD206 antibodies (Proteintech).

### Statistical Analysis

Sample size estimation was based on previous results from comparable studies, assuming 80% power at a significance level of 0.05. Specific details on how many independent biological samples or mice were included in an experiment or how many experiments were repeated independently are provided in the corresponding figure legends. Normality was analyzed using the Shapiro–Wilk test, and variance was analyzed using the F‐test for two groups or Bartlett's test for multiple comparisons. When the data passed the normality test, two groups of data with equal variance were analyzed using the 2‐tailed Student's *t*‐test, and those with unequal variance were evaluated using the Welch test. Multiple comparisons of data with normality and equal variance were performed using one‐way ANOVA. When the data did not pass the normality test, they were analyzed using the Mann–Whitney test for 2 group comparison or the Kruskal–Wallis test for multiple comparisons. When over two groups of two categorical variables passed the normality and equal variance tests, 2‐way ANOVA was employed. Post hoc tests were performed using the Bonferroni test. The data tested by parametric tests are shown as the mean and SEM, and the data tested by nonparametric tests are represented as medians with 95% confidence limits. Statistical significance was set at *p* < 0.05. SPSS software was used to perform all statistical analyses.

### Study Approval

Animal use and care were approved by the Animal Care and Use Ethics Committee of Shandong Provincial Hospital (approval no. 2021‐272).

## Conflict of Interest

The authors declare no conflict of interest.

## Supporting information



Supporting Information

## Data Availability

The data that support the findings of this study are available from the corresponding author upon reasonable request.
