# Supplementary material for: Nanoparticle‐Modified Nanofibers Induce Ferroptosis and Stimulate Antitumor Immunity for Melanoma Therapy
Source: Adv Sci (Weinh). 2025 Oct 20;13(1):e08753. doi: 10.1002/advs.202508753 (PMC12767067; doi:10.1002/advs.202508753)
Supplement: Supplementary file 1 — Supporting Information [file ADVS-13-e08753-s001.docx]

**Supporting Information for**

**Nanoparticle-Modified Nanofibers Induce Ferroptosis and Stimulate Antitumor Immunity for Melanoma Therapy**

*Mingyang Li^†^, Rongrong Li^†^, Baotong Xu, Yuhan Lian, Chenyu Yang, Jingwen Li, Junhao Liang, Linhan Ding, Dongsheng Zhang^a^, Jing Guo^*^, Xiao Fu^*^*

M. Li, R. Li, B. Xu, Y. Lian, C. Yang, J. Li, J. Liang, L. Ding, D. Zhang, X. Fu

Department of Stomatology, Shandong Provincial Hospital Affiliated to Shandong First Medical University, 324 Jingwu Road, 250021 Jinan, Shandong, China.

J. Guo

Department of Dermatology, Shandong Provincial Hospital Affiliated to Shandong First Medical University, No.324 Jingwuweiqi Road, Huaiyin District, Jinan, Shandong Province 250021, P.R. China

X. Fu

School of Stomatology, Shandong First Medical University and Shandong Academy of Medical Sciences, Jinan, Shandong 250021, P.R. China

Corresponding Author:

Xiao Fu, PhD

E-mail addresses: fuxiao@sdfmu.edu.cn

Jing Guo, Ph.D

E-mail addresses: guojing@email.sdfmu.edu.cn

These authors contributed equally: Mingyang Li, Rongrong Li

**This file includes:**

Figures. S1-S15

**
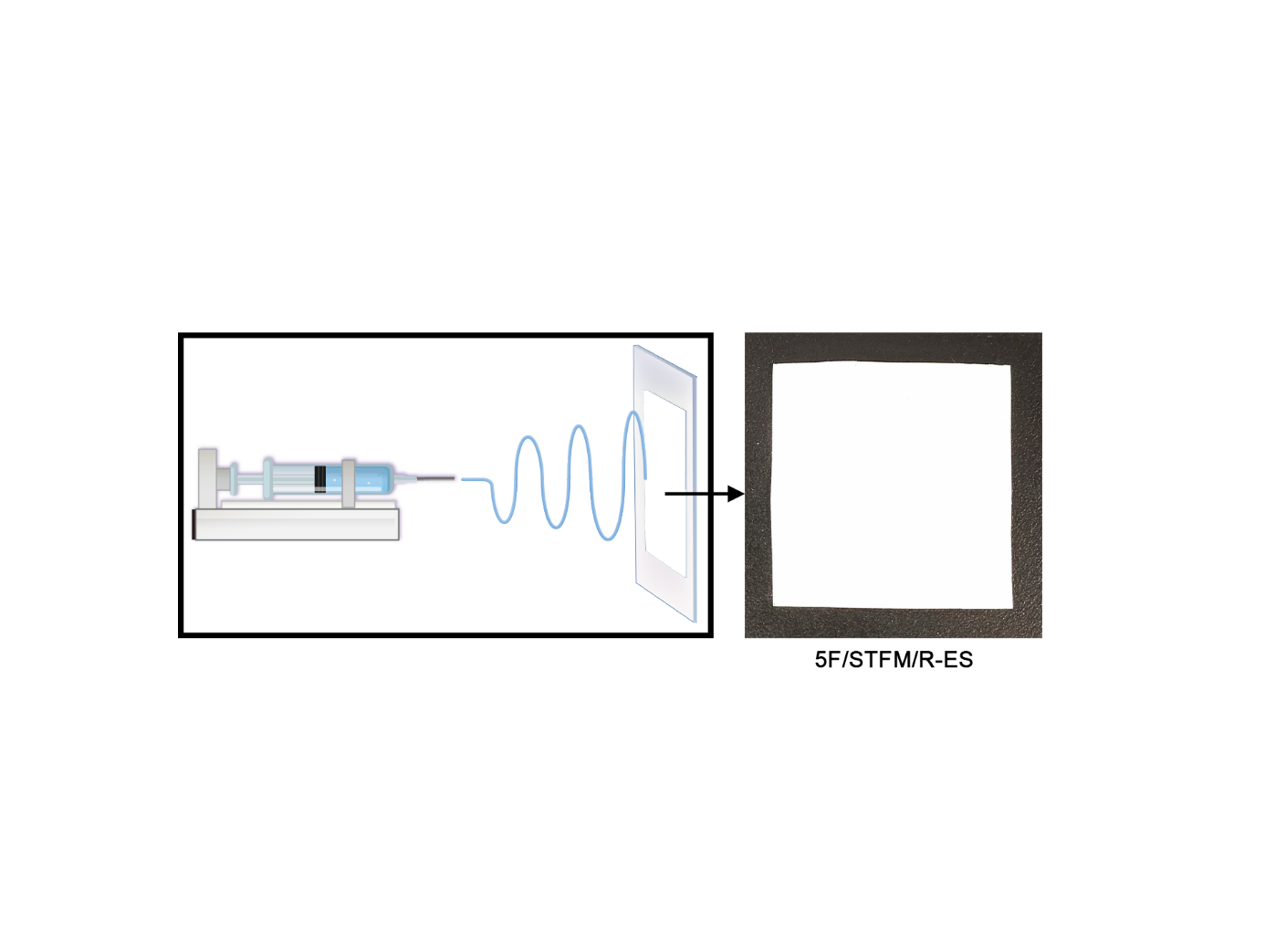
Fig S1**

**Fig S1** Schematic showing the synthesis and collection of 5F-STFM/R-ES. Following synthesis, the nanofibers (5F-STFM/R-ES) were deposited onto the collection plate to form a thin film.


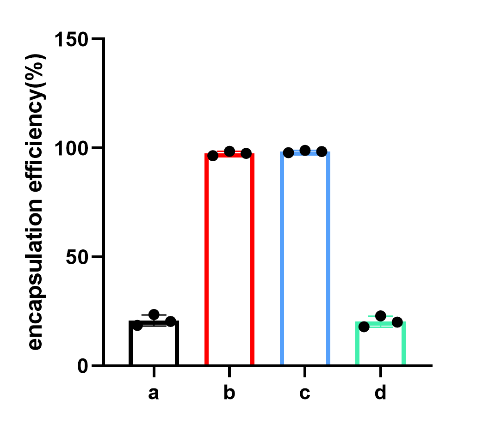
**
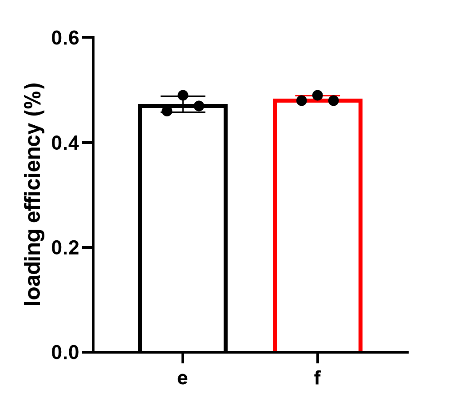
Fig S2**

**Fig S2** The encapsulation efficiency and loading efficiency of R848 and 5F in 5F/STFM/R-ES. a: encapsulation efficiency of R848 in STFM/R (n=3 experiments). b: encapsulation efficiency of STFM/R in 5F/STFM/R-ES (n=3 experiments). c: encapsulation efficiency of 5F in 5F/STFM/R-ES (n=3 experiments). d: Total encapsulation efficiency of R848 in 5F/STFM/R-ES (n=3 experiments). e: loading efficiency of R848 in 5F/STFM/R-ES (n=3 experiments). f: loading efficiency of 5F in 5F/STFM/R-ES (n=3 experiments).

**
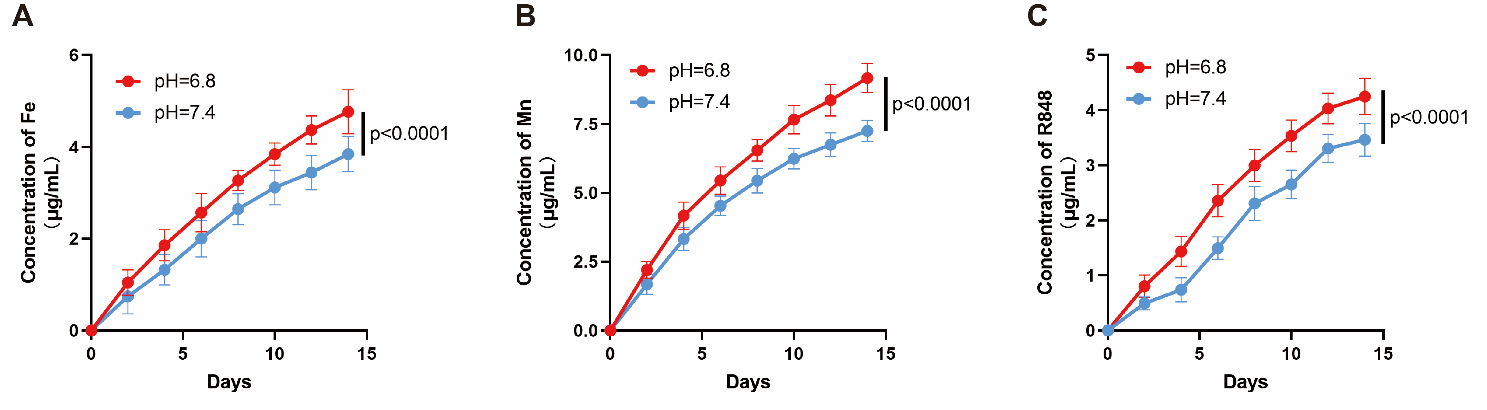
Fig S3**

**Fig S3** The release of iron, manganese, and R848 from 5F/STFM/R-ES. **A, B** The concentrations of iron and manganese were quantified using ICP (n=3 experiments). **C** The concentration of R848 was determined via HPLC (n=3 experiments). Data tested by parametric tests are presented as mean ± SEM. P values were determined by two-way ANOVA test

**
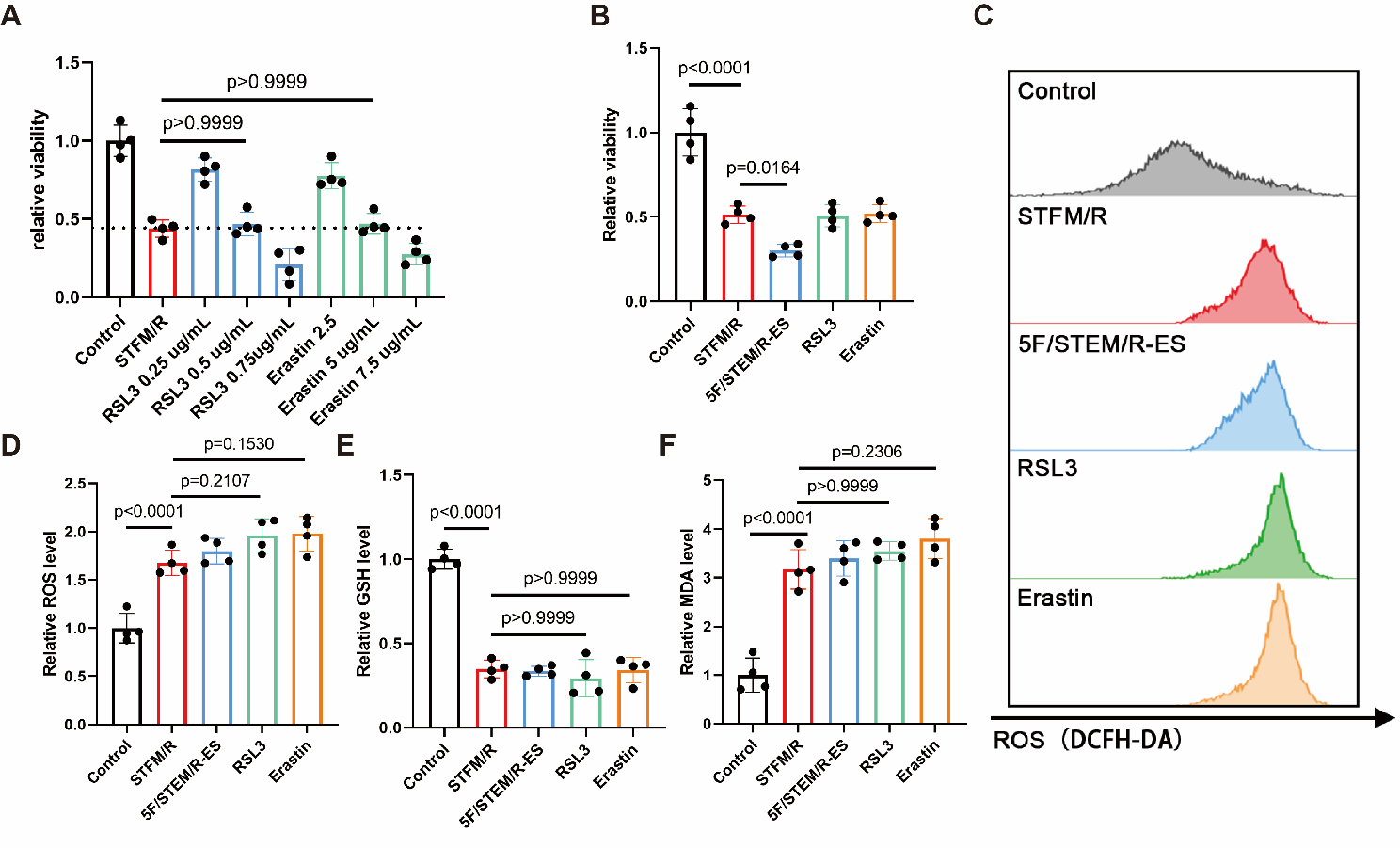
Fig S4**

**Fig S4** The assessment of STFM/R and 5F/STFM/R-ES for ferroptosis induing ability as compared with positive control group (0.5 μg/mL RSL3 or 5 μg/mL Erastin in B-F). Concentration of Fe in the incubation system of STFM/R group and 5F/STFM/R-ES group was 19.9 μg/mL. **A-B** CCK assay results showed the cell viability in each group (n=4 experiments). **C, D** ROS level in B16-F10 cells were measured by DCFH-DA (n=4 experiments). **E** The results showing the relative GSH level in B16-F10 cells (n=4 experiments). **F** The results showing the relative MDA level in B16-F10 cells (n=4 experiments). Data tested by parametric tests are presented as mean ± SEM. P values were determined by one-way ANOVA with post hoc Bonferroni test.

**
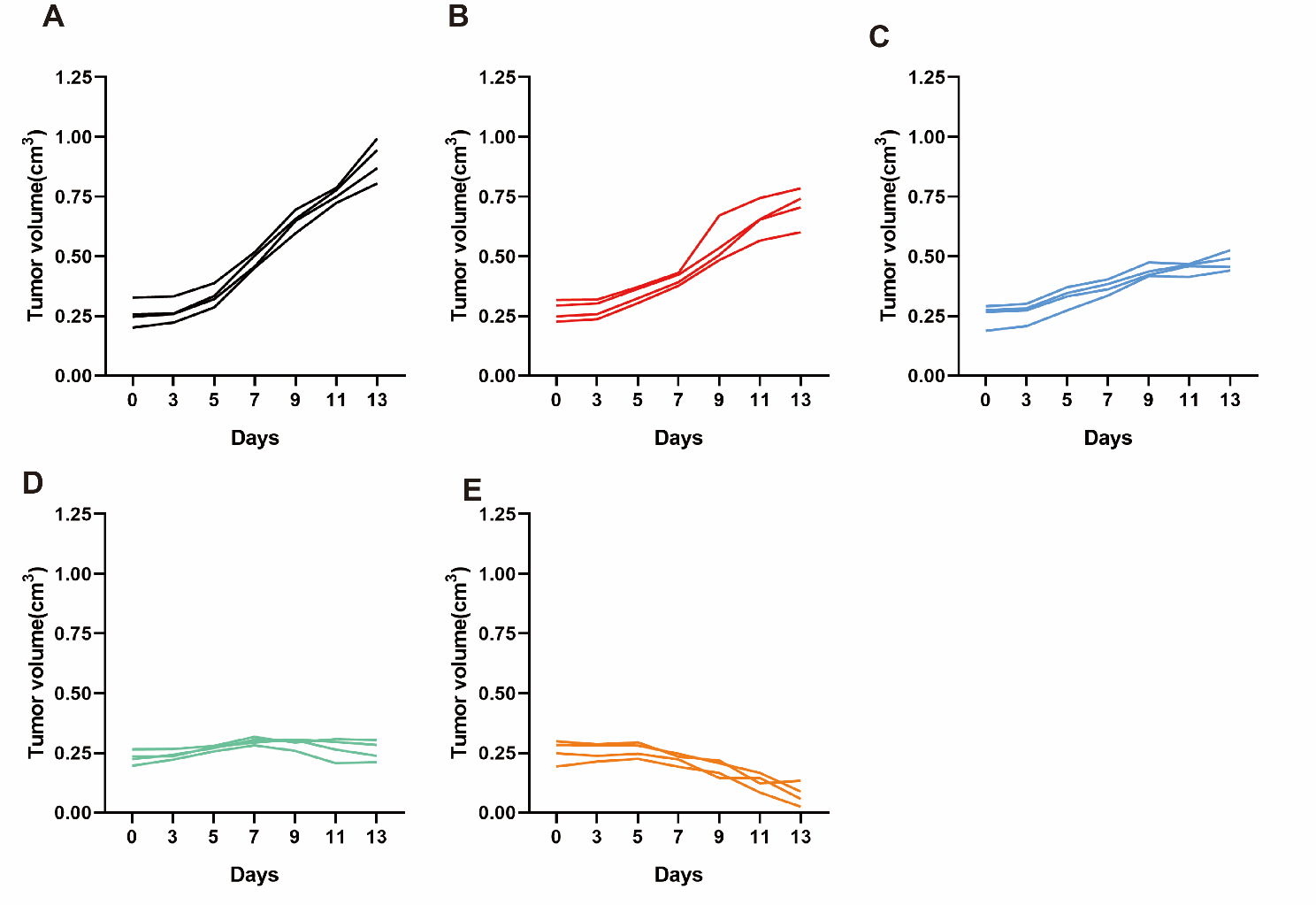
Fig S5**

**Fig S5** Tumor volume changes in each group with different treatments: control (A), 5-FU (B), 5F-ES (C), 5F/STFM-ES (D), 5F/STFM/R-ES (E) (n=4 mice).

**
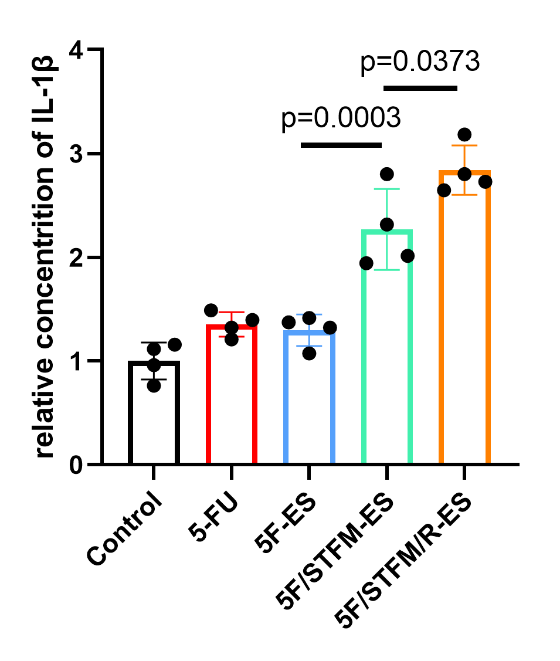
Fig S6**

**Fig S6** The ELISA results showing the release of IL-1β in melanoma (n=4 mice).

**Fig S7**

**
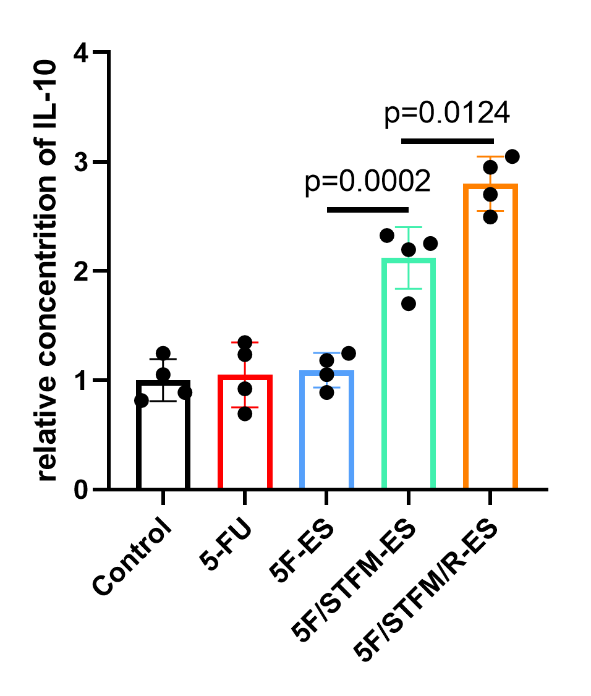
**

**Fig S7** The ELISA results showing the release of IL-6 in melanoma (n=4 mice).

**Fig S8**

**
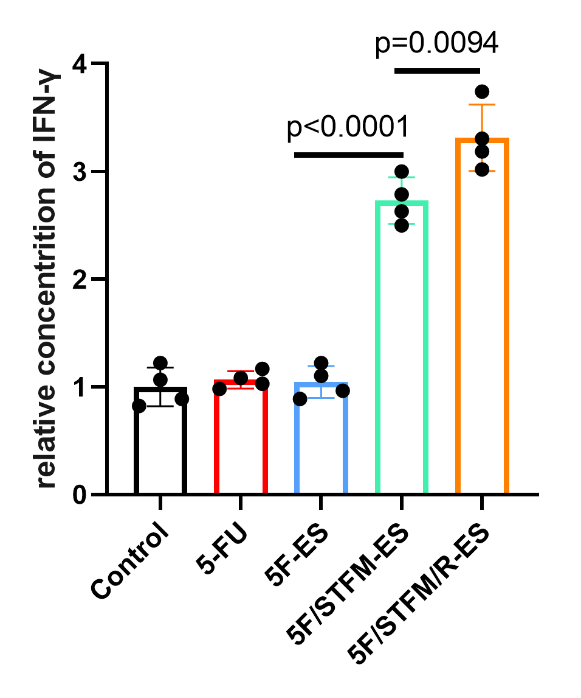
**

**Fig S8** The ELISA results showing the release of IFN-γ in melanoma (n=4 mice).

**Fig S9**

**
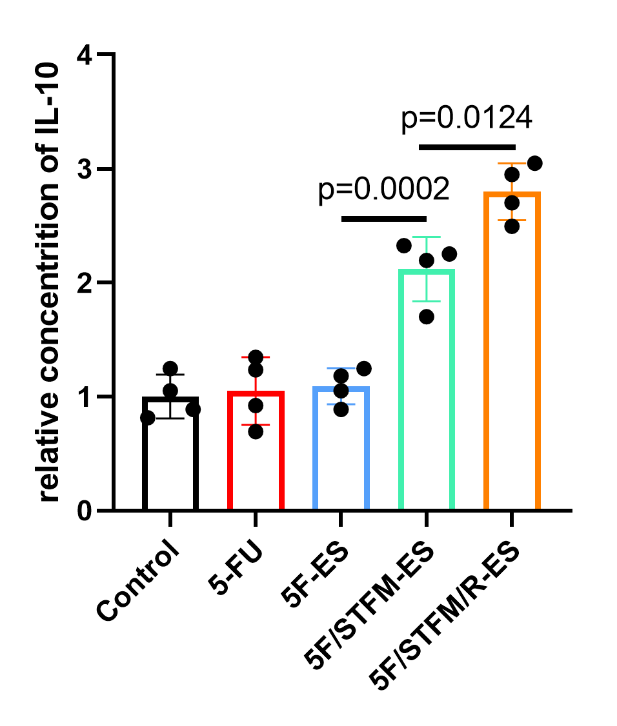
**

**Fig S9** The ELISA results showing the release of IL-10 in melanoma (n=4 mice).

**Fig S10**


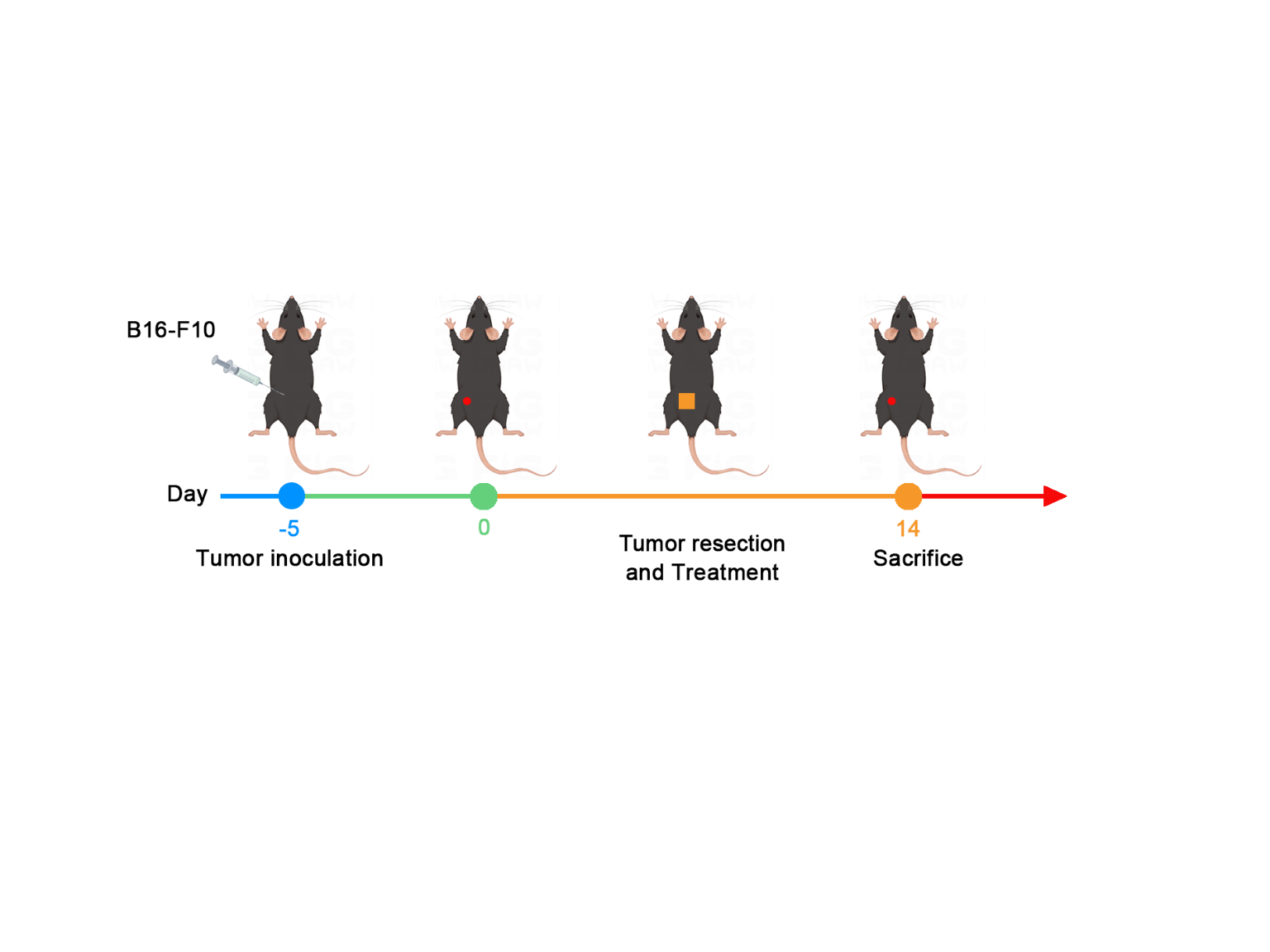


**Fig S10** Schematic illustrating the establishment and treatment of melanoma recurrence models.

**Fig S11**

**
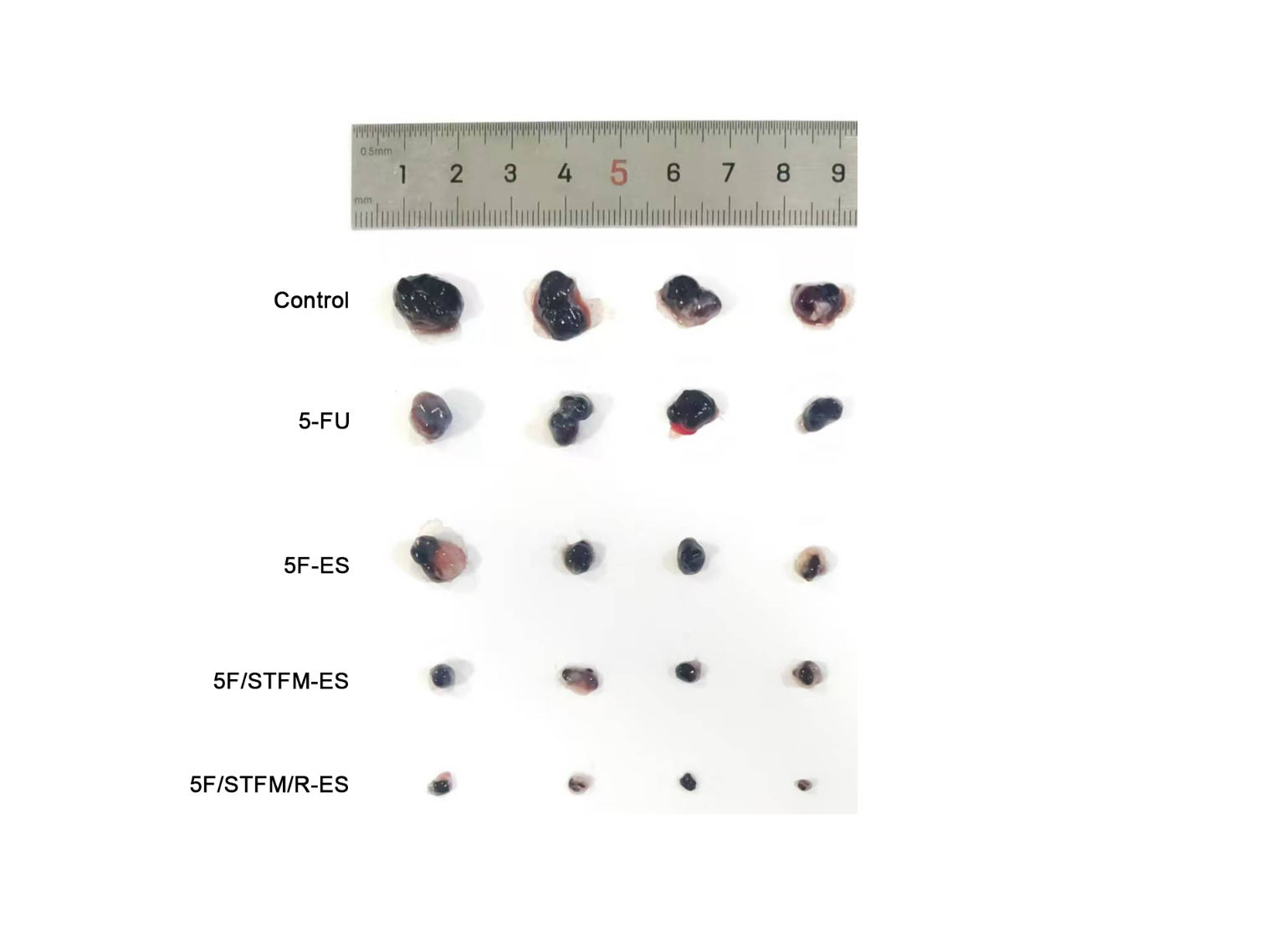
**

**Fig S11** Images displaying the melanomas of the mice with indicated treatment in the tumor recurrence experiments (n=4 mice).

**Fig S12**

**
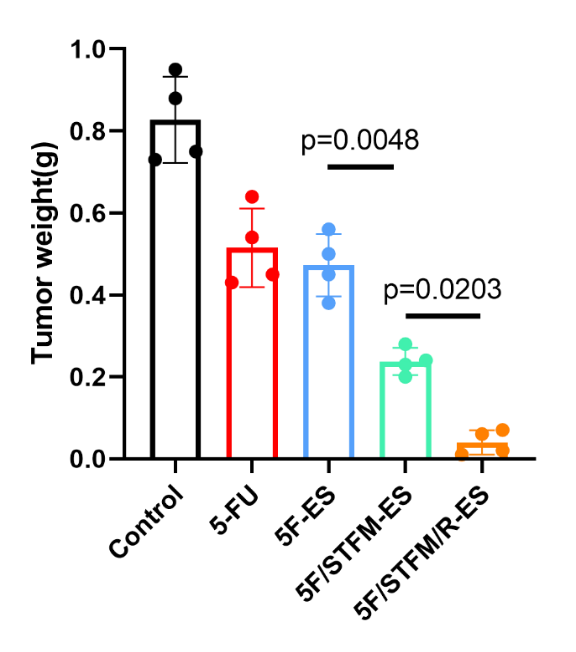
**

**Fig S12** The tumor weight in each group with different treatments in the tumor recurrence experiments (n=4 mice).

**
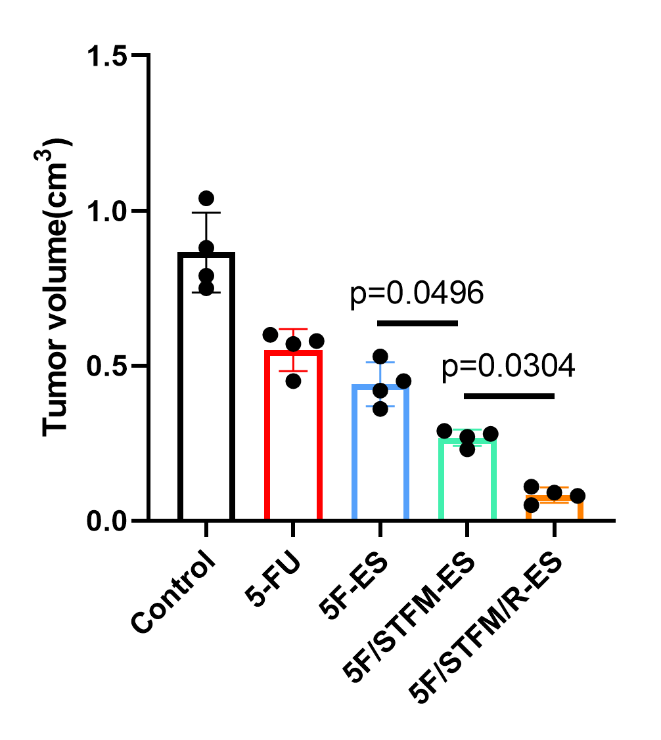
Fig S13**

**Fig S13** The tumor volume in each group with different treatments in the tumor recurrence experiments (n=4 mice).

**Fig S14**

**
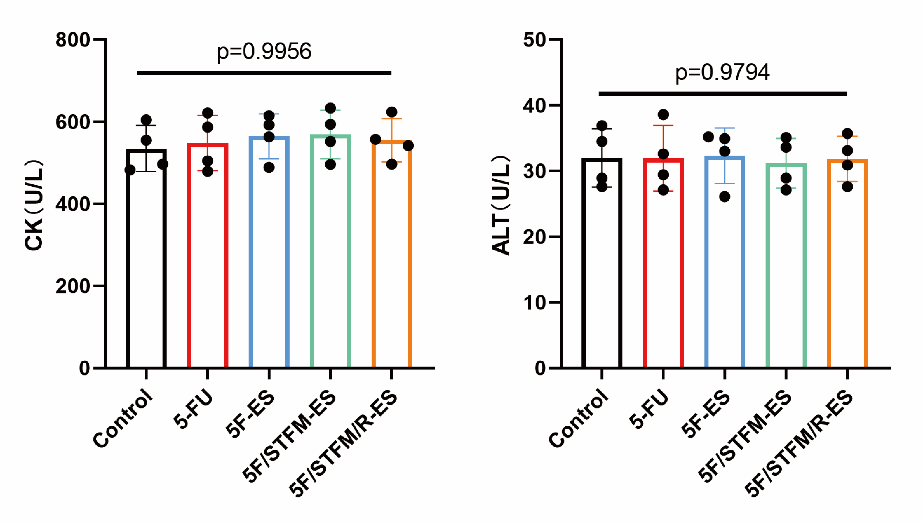

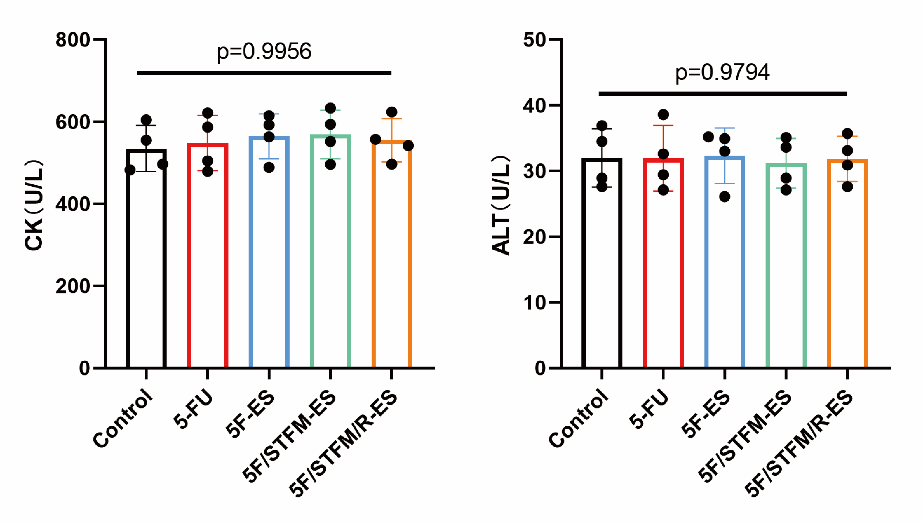
**

**Fig S14** Serum biochemical indicators analysis of CK and ALT (n=4 mice).

**
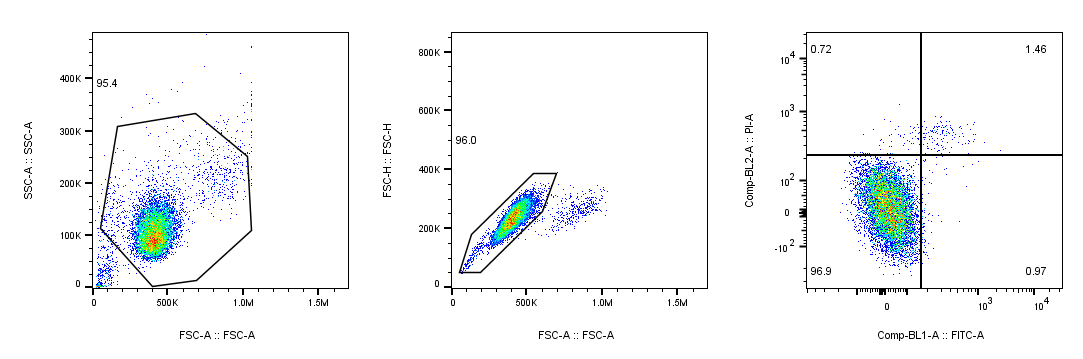
Fig S15**

**Fig S15** The gating strategy for apoptosis detection.
